# NK Cell Activation by Platinum Boosts Immunotherapy in HR^+^/HER2^−^ Breast Cancer

**DOI:** 10.1002/advs.202518978

**Published:** 2026-02-15

**Authors:** Yi‐Yu Chen, Yi‐Fan Zhou, Xuan Qi, Ya‐Xin Zhao, Tong Fu, Xi Jin, Minhong Shen, Yi‐Zhou Jiang, Zhi‐Ming Shao

**Affiliations:** ^1^ Key Laboratory of Breast Cancer in Shanghai Department of Breast Surgery Fudan University Shanghai Cancer Center; Department of Oncology Shanghai Medical College Fudan University Shanghai China; ^2^ Shanghai Academy of Natural Sciences (SANS) Fudan University Shanghai China

**Keywords:** HR^+^/HER2^−^ breast cancer, immune checkpoint inhibitors, immunotherapy, NK cells, platinum therapy

## Abstract

Immunotherapy revolutionizes cancer therapeutics but shows limited efficacy in hormone receptor‐positive (HR^+^)/human epidermal growth factor receptor 2‐negative (HER2^−^) breast cancer. By leveraging large‐scale multi‐omics and single‐cell RNA sequencing (scRNA‐seq), we characterize the tumor microenvironment of HR^+^/HER2^−^ breast cancer, revealing that an abundance of activated natural killer (NK) cells correlates with favorable anti‐PD‐(L)1 responses. Our preclinical models demonstrate that immunotherapy enhances NK cell cytotoxicity and immunomodulatory functions, thereby impeding tumor growth. Furthermore, drug screening of cell lines and patient‐derived tumors reveals that platinum enhances NK cell cytotoxicity, potentially via the NF‐κB pathway, creating synergy with immunotherapy. Consistent with these findings, a clinical cohort analysis shows an increased proportion of activated NK cells in tumors following platinum‐based chemotherapy. Collectively, our study establishes the critical role of NK cells in mediating immunotherapy response in HR^+^/HER2^−^ breast cancer and uncovers a novel mechanism whereby platinum agents augment immunotherapeutic efficacy, offering a promising combination strategy.

## Graphical abstract

Multi‐omics and single‐cell analyses identify activated NK cells as key mediators of anti‐PD‐(L)1 response in HR^+^/HER2^−^ breast cancer. Platinum chemotherapy enhances NK cell cytotoxicity through NF‐κB–associated signaling, synergizing with immunotherapy and providing a rationale for combination treatment.

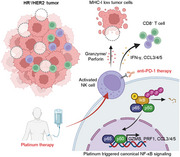

## 1 Introduction

Breast cancer is the most frequently diagnosed cancer in women worldwide and remains a leading cause of cancer‐related mortality, particularly among younger women. Globally, more than 2 million new cases and over 6 00 000 deaths were reported in 2021 [1, 2]. Breast cancer represents a diverse range of subtypes classified by the expression of hormone receptors (HRs: estrogen receptor (ER) and/or progesterone receptor (PR)) and the status of the human epidermal growth factor receptor 2 (HER2) [3, 4]. HR‐positive/HER2‐negative (HR^+^/HER2^−^) breast cancer is the most common molecular subtype of breast cancer, accounting for 60%–70% of all breast cancers, and is considered a relatively “indolent” subtype due to its high endocrine responsiveness [5]. Nonetheless, some HR^+^/HER2^−^ patients develop resistance to standard hormonal treatments, which correlates with an increased risk of long‐term recurrence [6, 7]. To address this issue, various target‐based precision therapies, including cyclin‐dependent kinase 4 and 6 (CDK4/CDK6) inhibitors, have been introduced to enhance the survival rates of HR^+^/HER2^−^ patients [8, 9, 10, 11]. Despite the precision of these drugs in targeting cancer cells, their efficacy is constrained by the limited investigation of the landscape of druggable targets and relevant biomarkers, considering tumor heterogeneity.

Immune checkpoint inhibitors (ICIs) targeting programmed death‐1 (PD‐1) and programmed death‐ligand 1 (PD‐L1) have revolutionized the treatment of solid tumors [12]. The strongest evidence for the efficacy of ICIs in breast cancer has been observed in triple‐negative breast cancer (TNBC), where the tumor microenvironment (TME) has been extensively investigated. Multiple clinical trials have demonstrated the effectiveness of ICIs in both neoadjuvant and advanced treatment settings [13, 14]. In contrast to TNBC, HR^+^/HER2^−^ breast cancer often lacks immune cell infiltration and expression of major histocompatibility complex (MHC) class I molecules and ICI targets, including PD‐1 and PD‐L1 [15, 16], posing a challenge for immunotherapy in HR^+^/HER2^−^ breast cancer. Recently, we established a large‐scale multi‐omics cohort of patients with HR^+^/HER2^−^ breast cancer, revealing an immunogenic subgroup characterized by a high level of immune cell infiltration [17]. This finding compels us to focus more closely on the microenvironmental landscape and immunotherapy responses in HR^+^/HER2^−^ breast cancer, which is a complex and evolving area of investigation. For example, the phase Ib KEYNOTE‐028 trial reported an objective response rate of 12% in advanced HR^+^/HER2^−^ patients with PD‐L1‐positive tumors treated solely with pembrolizumab [18]. In a phase II GIADA trial, a pathological complete response (pCR) rate of 16.3% was achieved with neoadjuvant sequential anthracyclines and nivolumab for luminal B‐like HR^+^/HER2^−^ cancer [19]. Recently, a phase II I‐SPY2 platform trial demonstrated that the addition of pembrolizumab or durvalumab to chemotherapy increased the pCR rate in high‐risk HR^+^/HER2^−^ early cases [20, 21]. Although these studies preliminarily revealed the potential of immunotherapy in HR^+^/HER2^−^ breast cancer, biomarkers for stratifying patients who will benefit from immunotherapy remain to be determined. To address this issue, we focused on delineating the immune context within HR^+^/HER2^−^ breast cancer and developing predictive biomarkers for immunotherapy response.

In this study, we profiled the microenvironment of HR^+^/HER2^−^ breast cancer and performed single‐cell RNA sequencing (scRNA‐seq) of baseline samples from patients before receiving immunotherapy. Our analysis revealed that an abundance of activated natural killer (NK) cells was predictive of favorable treatment efficacy. Mechanistic investigations subsequently confirmed that NK cells potentiate the response to anti‐PD‐(L)1 therapy. Critically, we discovered that platinum‐based agents could activate these cells to augment the therapeutic effect, providing a strong rationale for a novel combination strategy in HR^+^/HER2^−^ breast cancer.

## 2 Results

### 2.1 Microenvironment Phenotypes of HR^+^/HER2^−^ Breast Cancer

**FIGURE 1:**
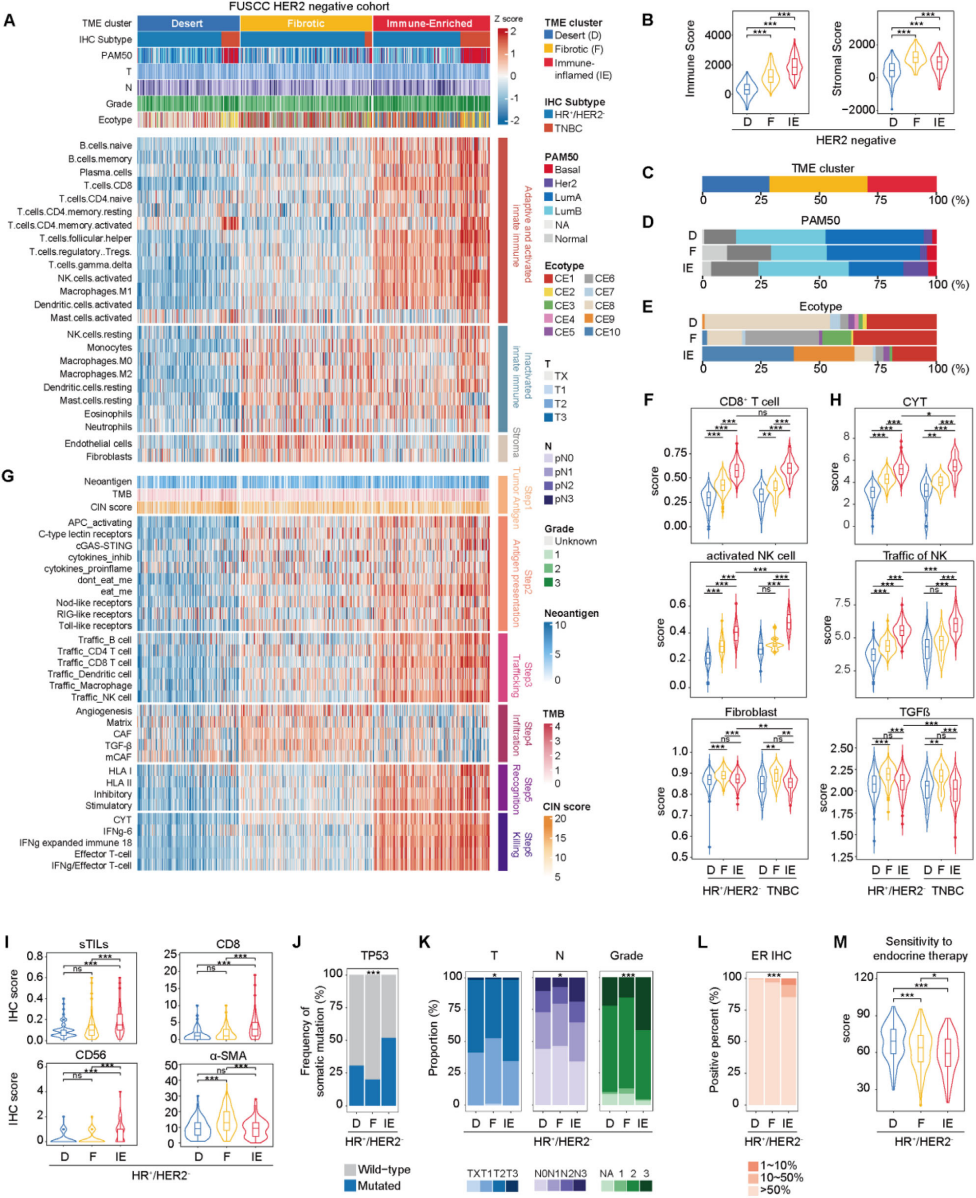
Tumor microenvironment subtyping in HR^+^/HER2^−^ breast cancer. (A) K‐means clustering of HER2‐negative breast cancer based on the enrichment scores of 24 microenvironment cell subsets calculated by ssGSEA. (B) The stromal and immune scores among TME subtypes. The central line of each boxplot within violin plots represents the median value, and the lower and upper hinges indicate the 25th and 75th percentiles, respectively. *P* values were from the two‐sided unpaired Wilcoxon test. (C) Distribution of TME subtypes among HR^+^/HER2^−^ breast cancer. (D) Distribution of PAM50 subtypes among the clusters in HR^+^/HER2^−^ breast cancer. (E) Distribution of Ecotype subtypes among the clusters in HR^+^/HER2^−^ breast cancer. (F) Signature scores of CD8^+^ T cells, activated NK cells, and fibroblasts among clusters and clinical subtypes. The central line of each boxplot within violin plots represents the median value, and the lower and upper hinges indicate the 25th and 75th percentiles, respectively. *P* values were from the two‐sided unpaired Wilcoxon test. (G) The landscape of six steps in the Cancer‐Immunity Cycle in HER2‐negative tumors. (H) Signature scores of the CYT score, chemotaxis of NK cells, and TGF‐β pathway among clusters and clinical subtypes. The central line of each boxplot within violin plots represents the median value, and the lower and upper hinges indicate the 25th and 75th percentiles, respectively. *P* values were from the two‐sided unpaired Wilcoxon test. (I) Pathologic scores of sTILs and IHC scores of CD8, CD56, and α‐SMA among clusters. The central line of each boxplot within violin plots represents the median value, and the lower and upper hinges indicate the 25th and 75th percentiles, respectively. *P* values were from the two‐sided unpaired Wilcoxon test. (J) The association between somatic mutations and HR^+^/HER2^−^ TME phenotypes. *P* values were from the Fisher's exact test test. (K) Comparison of clinicopathologic characteristics among the clusters in HR^+^/HER2^−^ breast cancer. *P* values were from chi‐square test. (L) Comparison of the IHC score of ER among the clusters in HR^+^/HER2^−^ breast cancer. *p* value was from the chi‐square test. (M) Signature scores of sensitivity to endocrine therapy among the clusters in HR^+^/HER2^−^ breast cancer. The central line of each boxplot within violin plots represents the median value, and the lower and upper hinges indicate the 25th and 75th percentiles, respectively. *P* values were from the two‐sided unpaired Wilcoxon test. See also Figures S1 and S2. FUSCC, Fudan University Shanghai Cancer Center; HER2, human epidermal growth factor receptor 2; TNBC, triple‐negative breast cancer; ER, estrogen receptor; HR, hormone receptor; IHC, immunohistochemistry; TME, tumor microenvironment; TMB, tumor mutation burden; CIN, chromosomal instability; D, Desert; F, Fibrotic; IE, Immune‐Enriched.

We investigated the TME of 672 HER2‐negative breast cancer patients from our previously established Chinese breast cancer cohorts [17, 22]. This cohort included both HR^+^/HER2^−^ and TNBC patients, facilitating a comparative analysis. Multi‐omics data were available for this cohort, including transcriptome sequencing for all 672 patients, whole exome sequencing (WES) of matched tumor‐blood pairs for 576 patients, and copy number alteration (CNA) data from tumor tissue for 480 patients (Figure S1A). Using transcriptomic data to infer the abundance of 24 microenvironment cell subsets [23], we performed k‐means clustering and classified all 672 tumors into three distinct TME phenotypes: ‘Desert’ (D), characterized by a general scarcity of microenvironmental cells; ‘Fibrotic’ (F), enriched with stromal cells and inactive innate immune cells; and ‘Immune‐Enriched’ (IE), defined by a high abundance of adaptive and activated innate immune cells, indicative of an inflamed TME (Figure 1A,B). The robustness of these TME classifications was validated using alternative computational methods (CIBERSORT, xCell) and an independent TCGA breast cancer cohort (Figure S1B–D). Within the HR^+^/HER2^−^ subgroup, the TME phenotypes were distributed as subtypes D (28.5%), F (42.0%), and IE (29.5%) (Figure 1C). When correlated with the PAM50 intrinsic subtypes, the IE subtype was associated with more aggressive molecular features, including a higher proportion of HER2‐enriched tumors and a lower proportion of Luminal A tumors (Figure 1D). Furthermore, the IE subtype exhibited a higher prevalence of cellular ecotypes 9 (CE9) and 10 (CE10) [24] (Figure 1E).

We next delineated the cellular composition of these TME subtypes, revealing a paradoxical state within the HR^+^/HER2^−^ IE subtype. Although these tumors were significantly enriched in cytotoxic lymphocytes (CD8^+^ T cells and NK cells) relative to their Desert and Fibrotic counterparts, they displayed a less potent immune profile than the TNBC IE subtype, characterized by a lower proportion of NK cells and a more pronounced fibrotic phenotype (Figure 1F and Figure S1E, F). Beyond cytotoxic lymphocytes, myeloid populations also exhibited distinct distributions across TME subtypes. The D subtype was characterized by an enrichment of activated mast cells, whereas the F subtype showed increased neutrophils, M2 macrophages, and resting mast cells. In contrast, the IE subtype was preferentially enriched in M1 macrophages and dendritic cells, consistent with an immune‐activated microenvironment (Figure S1G).

Analysis of the cancer‐immunity cycle revealed key functional disparities (Figure 1G). The mutational analysis of HR^+^/HER2^−^ breast cancer subtypes revealed distinct patterns. There were no significant differences in neoantigen load among the three groups (Figure S1H). However, the IE subtype exhibited a significantly higher mutation load compared to the D and F subtypes (Figure S1H). Regarding chromosomal instability (CIN), the D subtype displayed the highest CIN score, which was significantly higher than both the F and IE subtypes (Figure S1H). Processes related to cytotoxic cell chemotaxis and killing were significantly upregulated in the HR^+^/HER2^−^ IE subtype compared to other HR^+^/HER2^−^ subtypes yet remained less active than in the TNBC IE subtype (Figure 1H). Conversely, immune evasion pathways, particularly TGF‐β signaling associated with cancer‐associated fibroblasts, were more prominent in the HR^+^/HER2^−^ IE subtype than in their TNBC IE counterparts (Figure 1H). These findings indicate that despite an increased immune cell presence in HR^+^/HER2^−^ IE tumors, they maintained a relatively colder and more fibrotic profile than TNBC IE tumors.

To validate these transcriptomic classifications, histopathological and immunohistochemical (IHC) analyses were performed. Quantification of stromal tumor‐infiltrating lymphocytes (sTILs) confirmed a significantly higher lymphocyte density in the HR^+^/HER2^−^ IE subtype (Figure 1I). Moreover, IHC staining for CD8a, CD56, and α‐SMA verified the increased abundance of CD8^+^ T and NK cells in the IE subtype and the enrichment of fibroblasts in the F subtype (Figure 1I and Figure S2A–C).

Upstream genomic analyses were performed to uncover the genomic underpinnings of these TME phenotypes. At the mutational level, TP53 mutations were significantly enriched in the HR^+^/HER2^−^ IE subtype (Figure 1J). At the copy number level, the IE subtype was characterized by recurrent gain/amplification of 8q23.3 and deletion/loss of 5q11.2 and 5q22.2 (Figure S2D).

Finally, we investigated the clinical significance of TME subtypes. In the HR^+^/HER2^−^ cohort, the IE subtype was strongly associated with adverse clinicopathological features, including larger tumor size, positive lymph node status, and higher histological grade (Figure 1K), which were not observed in TNBC (Figure S2E). The IE subtype was also enriched in tumors with low ER expression (<10%), and demonstrated the lowest predicted sensitivity to endocrine therapy (Figure 1L,M). In summary, the ‘Immune‐Enriched’ phenotype within HR^+^/HER2^−^ breast cancer identifies a clinically high‐risk patient population that is less likely to benefit from standard endocrine therapy. This distinct biology underscores the potential of exploiting the immune‐infiltrated TME with alternative strategies, such as immunotherapy.

### 2.2 Activated NK Cells are Associated with Favorable Response to Anti‐PD‐(L)1 Immunotherapy in HR^+^/HER2^−^ Breast Cancer

**FIGURE 2:**
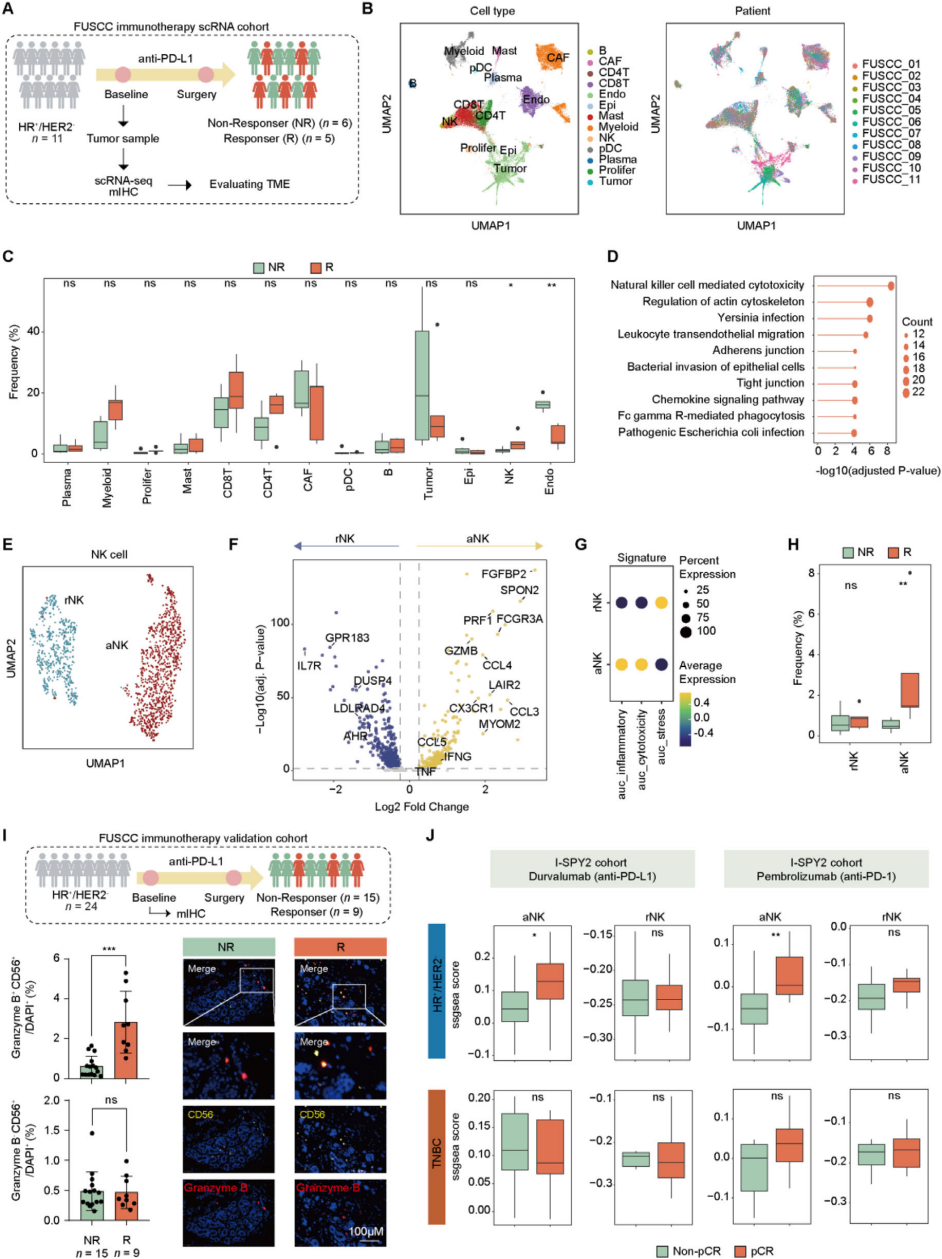
Activated NK cells were associated with favorable response to anti‐PD‐(L)1 therapy in HR^+^/HER2^−^ breast cancer. (A) The overview of the FUSCC HR^+^/HER2^−^ immunotherapy scRNA cohort. (B) Uniform manifold approximation and projection (UMAP) visualization of 69784 cells from 11 HR^+^/HER2^−^ samples, colored by major cell lineages and their patient of origin. (C) Abundance differences of major cell lineages between the different efficacy groups. The central line of each boxplot represents the median value, and the lower and upper hinges indicate the 25th and 75th percentiles, respectively. *P* values were from the two‐sided unpaired Wilcoxon test. (D) KEGG pathway enrichment analysis of genes upregulated in NK cells from responders. (E) UMAP plot showing the sub‐clustering of NK cells into resting (rNK) and activated (aNK) subsets. (F) Differences in the expression of key marker genes for the resting and activated NK cells. (G) Differences in the functional signatures for the resting and activated NK cells. (H) Abundance differences of NK cell subsets between the different efficacy groups. The central line of each boxplot represents the median value, and the lower and upper hinges indicate the 25th and 75th percentiles, respectively. *P* values were from the two‐sided unpaired Wilcoxon test. (I) mIHC analysis of Granzyme B^+^ CD56^+^ and Granzyme B^−^ CD56^+^ NK cells in tumor sections from the FUSCC immunotherapy validation cohort. Data are presented as mean ± SD. Two‐side Wilcoxon rank‐sum test. (J) Comparison of single‐sample gene set enrichment analysis (ssGSEA) scores for activated and resting NK cell signatures in patients from the I‐SPY2 cohort. The central line of each boxplot represents the median value, and the lower and upper hinges indicate the 25th and 75th percentiles, respectively. *P* values were from the two‐sided unpaired Wilcoxon test. See also Figure S3. R, responders; NR, non‐responders; mIHC, multiplex immunohistochemistry; rNK, resting NK cells; aNK, activated NK cells.

To identify the predictors of response to neoadjuvant anti‐PD‐(L)1 immunotherapy, we performed scRNA‐seq on pre‐treatment biopsies from a prospective cohort of 11 patients with locally advanced HR^+^/HER2^−^ breast cancer. Following neoadjuvant anti‐PD‐L1 immunotherapy and surgery, the patients were stratified based on the Residual Cancer Burden (RCB) index into responders (R; RCB‐I/II, *n *= 5) and non‐responders (NR; RCB‐III, *n *= 6) (Figure 2A and Table S1).

Following quality filtering, our analysis yielded 69 784 high‐quality single cells annotated as distinct populations (Figure 2B and Figure S3A). We then analyzed the abundance of cellular populations among the different treatment groups. Interestingly, NK cells were enriched in responsive tumors, whereas endothelial cells were enriched in non‐responsive tumors (Figure 2C). To validate this finding, we performed STARTRAC analysis, which enables quantitative assessment of the distributional preference of clusters across different groups [25, 26]. Consistent with the results of direct frequency comparisons, STARTRAC analysis revealed a strong distributional preference of NK cells in responders and endothelial cells in non‐responders (Figure S3B). Given the clinical challenge of identifying patients with HR^+^/HER2^−^ breast cancer who are more likely to benefit from immunotherapy, we subsequently focused our analyses on elucidating the role of NK cells as potential mediators and biomarkers of favorable immunotherapy response.

To determine the functional state of NK cells, we compared their transcriptomic profiles between responders and non‐responders. NK cells from responders showed a significantly higher expression of key cytotoxic molecules (e.g., *GZMB* and *PRF1*) and chemokines critical for immune cell recruitment (e.g., *CCL3*, *CCL4*, and *CCL5*) (Figure S3C). Conversely, the transcriptomic profile of NK cells from non‐responders was characterized by an upregulation of immature marker IL7R and cyclic AMP response element modulator (CREM), which has been reported as a regulatory checkpoint in NK cells [27] (Figure S3C). KEGG pathway analysis confirmed that NK cells from responsive tumors were enriched in NK cell‐mediated cytotoxicity, chemokine signaling, and Fcγ receptor‐mediated phagocytic pathways (Figure 2D). This functional heterogeneity led us to sub‐cluster the NK population, identifying two distinct NK subsets: the resting NK cell (rNK) subset and the activated NK cell (aNK) subset (Figure 2E). The resting NK cell subset was marked by elevated IL7R expression and enrichment of stress‐related pathways. In contrast, the activated NK cell subset displayed high expression of effector molecules (e.g., *PRF1*, *GZMB*, and *IFNG*) and inflammatory chemokines (e.g., *CCL3*, *CCL4*, and *CCL5*), consistent with a robust cytotoxic and pro‐inflammatory transcriptional program (Figure 2F,G). Crucially, a quantitative comparison demonstrated that only the infiltration of the activated NK subset was significantly elevated in responders, whereas the abundance of the resting NK subset was comparable between the two efficacy groups (Figure 2H and Figure S3D). Given recent reports highlighting tumor‐promoting roles of immature NK cells in triple‐negative breast cancer [28], we further examined whether a similar NK subset exists in HR^+^/HER2^−^ tumors. Using established gene signatures, we identified immature NK cells predominantly within the resting NK compartment (Figure S3E). However, their abundance showed no significant correlation with immunotherapy response in our HR^+^/HER2^−^ cohort (Figure S3F).

To further evaluate the clinical value of activated NK cells in HR^+^/HER2^−^ breast cancer, we investigated the association between NK cells and response to anti‐PD‐(L)1 blockade in both internal and external cohorts. First, we performed multiplex immunohistochemistry (mIHC) to identify activated (Granzyme B^+^ CD56^+^) and resting (Granzyme B^−^ CD56^+^) NK cells in biopsies from our scRNA‐seq cohort, confirming the consistency between the scRNA‐seq data and the mIHC staining results (Figure S3G). Next, we applied this mIHC panel to an independent internal cohort consisting of pre‐treatment biopsies from 24 patients with early‐stage HR^+^/HER2^−^ breast cancer, who subsequently underwent neoadjuvant anti‐PD‐L1 therapy (Table S2). Consistent with our findings from the scRNA‐seq cohort, responders exhibited a significantly elevated abundance of activated NK cells compared to non‐responders, whereas the abundance of resting NK cells did not differ between the two groups (Figure 2I). Finally, we performed external validation using publicly available RNA‐seq and outcome data from the I‐SPY2 trial (NCT01042379), an adaptive platform trial designed to rapidly screen novel agents in the neoadjuvant setting for high‐risk early‐stage breast cancer [20, 21, 29]. The relevant immunotherapy arms included anti‐PD‐1 (pembrolizumab) or anti‐PD‐L1 (durvalumab) agents, combined with standard chemotherapy. By applying single‐sample gene set enrichment analysis (ssGSEA) to pre‐treatment tumor tissues, we calculated signatures for activated and resting NK cells. Intriguingly, the activated NK cell signature was strongly associated with immunotherapy benefits in HR^+^/HER2^−^ breast cancer. Remarkably, this association was absent in TNBC patients (Figure 2J). Collectively, these multi‐cohort findings suggest that the pre‐existing infiltration of activated NK cells is a potent predictive biomarker for the response to anti‐PD‐(L)1 therapy in HR^+^/HER2^−^ breast cancer.

### 2.3 HR^+^/HER2^−^ Breast Cancer is Vulnerable to NK Cell‐Mediated Cytotoxicity

**FIGURE 3:**
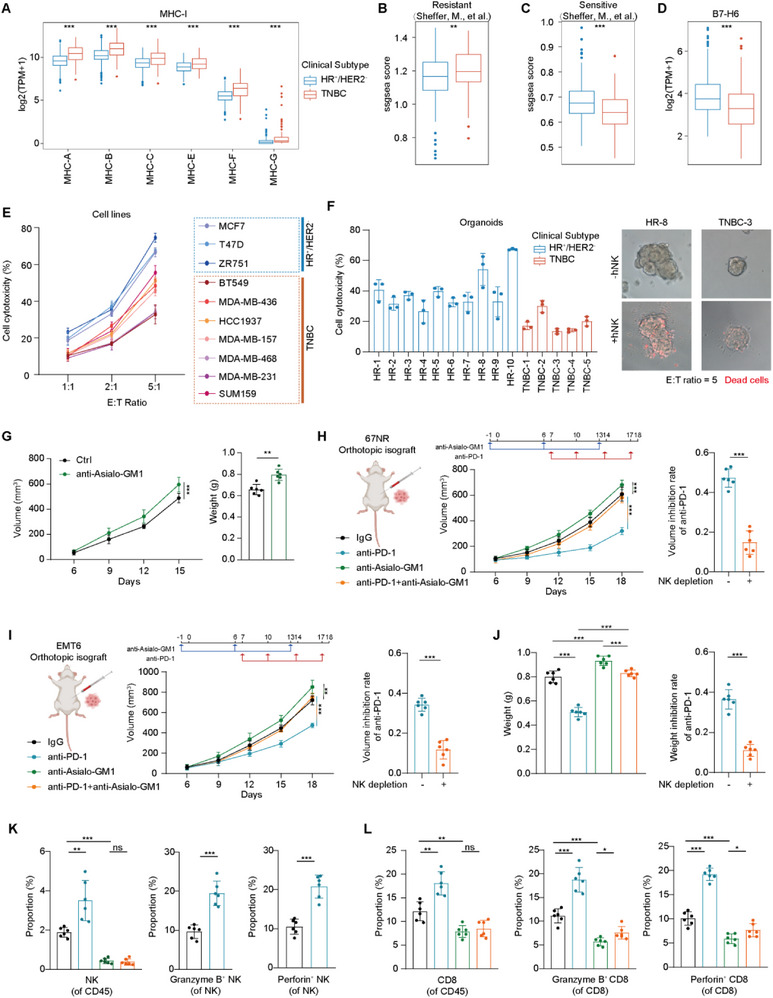
NK cells potentiate the efficacy of ICI therapy in HR^+^/HER2^−^ tumors. (A) Expression differences between classical and non‐classical MHC class I molecules in HR^+^/HER2^−^ and TNBC tumors. The central line of each boxplot represents the median value, and the lower and upper hinges indicate the 25th and 75th percentiles, respectively. *P* values were from the two‐sided unpaired Wilcoxon test. (B) Differences in the score of resistance to NK cell cytotoxicity between HR^+^/HER2^−^ and TNBC tumors. The central line of each boxplot represents the median value, and the lower and upper hinges indicate the 25th and 75th percentiles, respectively. *p* value was from the two‐sided unpaired Wilcoxon test. (C) Differences in the score of sensitivity to NK cell cytotoxicity between HR^+^/HER2^−^ and TNBC tumors. The central line of each boxplot represents the median value, and the lower and upper hinges indicate the 25th and 75th percentiles, respectively. *p* value was from the two‐sided unpaired Wilcoxon test. (D) Differences in the expression of B7‐H6 between HR^+^/HER2^−^ and TNBC tumors. The central line of each boxplot represents the median value, and the lower and upper hinges indicate the 25th and 75th percentiles, respectively. *p* value was from the two‐sided unpaired Wilcoxon test. (E) Cytotoxicity of NK cells against HR^+^/HER2^−^ and TNBC breast cancer cell lines. *n* = 3. (F) Cytotoxicity of NK cells against HR^+^/HER2^−^ and TNBC breast cancer organoids. Organoids were cultured alone or co‐cultured with human NK cells for 4 h. Quantitative data were obtained using NK cells isolated from three independent PBMC donors (*n* = 3). Representative images from one donor are shown on the right. (G) Growth curves and tumor weight at the endpoint of mice treated with anti‐Asialo‐GM1 in the 67NR orthotopic tumor model (*n* = 6 per group). Data are presented as mean ± SD. Two‐sided two‐way ANOVA for growth curves and two‐sided Student’s *t* test for tumor weight comparison at the endpoint. (H) Growth curves of mice treated with anti‐PD‐1 antibody and anti‐Asialo‐GM1 in the 67NR orthotopic tumor model (*n* = 6 per group). Data are presented as mean ± SD. Two‐sided two‐way ANOVA for growth curves and two‐sided Student's *t* test for tumor volume comparison at the endpoint. (I) Growth curves of mice treated with anti‐PD‐1 antibody and anti‐Asialo‐GM1 in the EMT6 orthotopic tumor model (*n* = 6 per group). Data are presented as mean ± SD. Two‐sided two‐way ANOVA for growth curves and two‐sided Student's *t* test for tumor volume comparison at the endpoint. (J) Tumor weight at the endpoint in mice treated with anti‐PD‐1 antibody and anti‐Asialo‐GM1 in the EMT6 orthotopic tumor model (*n* = 6 per group). Data are presented as mean ± SD. Two‐tailed unpaired Student's *t* test. (K) Infiltration and functional markers of NK cells measured by flow cytometry from EMT6 tumors. *n* = 6. Data are presented as mean ± SD. Two‐tailed unpaired Student's *t* test. (L) Infiltration and functional markers of CD8^+^ *T* cells measured by flow cytometry from EMT6 tumors. *n* = 6. Data are presented as mean ± SD. Two‐tailed unpaired Student's *t* test. See also Figures S4 and S5.

We explored the mechanisms underlying the enhanced benefits of ICI immunotherapy, facilitated by NK cells. While CD8^+^ T cell‐mediated cytotoxicity is contingent on MHC, NK cell‐mediated cytotoxicity is independent of MHC. In our multi‐omics cohort analysis, we investigated the expression of MHC class I molecules. Notably, HR^+^/HER2^−^ breast cancer exhibited a lower expression of MHC class I molecules than TNBC, accompanied by significantly reduced activity of MHC class I‐mediated pathways (Figure 3A and Figure S4A). Employing a published gene set [30], we next assessed NK cell cytotoxicity‐associated signatures. HR^+^/HER2^−^ tumors displayed a lower NK cytotoxicity‐resistant signature together with a higher NK cytotoxicity‐sensitive signature relative to TNBC (Figure 3B,C). In parallel, we examined the expression of ligands for NK cell‐activating receptors and found that NKG2D ligands, including MICA and MICB, as well as the NKp30 ligand B7‐H6, were expressed at higher levels in HR^+^/HER2^−^ tumors than in TNBC (Figure 3D and Figure S4B). Taken together, these findings suggest that HR^+^/HER2^−^ breast cancer might be susceptible to NK cell‐mediated cytotoxicity due to enhanced activating ligand expression and reduced inhibitory signaling.

To delve deeper into the role of NK cells in HR^+^/HER2^−^ breast cancer, we conducted a co‐culture killing assay involving NK and tumor cells. Compared with TNBC cell lines, HR^+^/HER2^−^ breast cancer cell lines were more vulnerable to NK cell‐mediated cytotoxicity (Figure 3E and Figure S4C). To assess tumor cell intrinsic susceptibility independent of stromal and immune components, we used patient‐derived organoid models to compare NK cell‐mediated cytotoxicity between HR^+^/HER2^−^ breast cancer and TNBC, yielding results consistent with those observed in cell lines (Figure 3F). Finally, we established an orthotopic isograft model using the HR^+^ murine breast cancer cell line 67NR and selectively depleted NK cells in vivo using anti‐Asialo‐GM1, which does not significantly affect major myeloid cells (Figure S4D). The results revealed a noteworthy acceleration in tumor growth upon NK cell depletion (Figure 3G). Taken together, these results confirm that HR^+^/HER2^−^ breast cancer is vulnerable to NK cell‐mediated cytotoxicity.

### 2.4 NK Cells Could Sensitize Immunotherapy in HR^+^/HER2^−^ Breast Cancer

Given that PD‐(L)1 signaling can induce NK cell exhaustion, we investigated whether PD‐(L)1 inhibitors could alleviate the exhaustion state of NK cells. Human NK cells were treated with anti‐PD‐1 antibody. We observed that markers of NK cell cytotoxicity, such as Granzyme B, CD107a, and Perforin, were significantly increased (Figure S5A,B). The co‐culture killing assay also confirmed that the cytotoxicity of NK cells against HR^+^/HER2^−^ cell lines was enhanced after treatment with anti‐PD‐1 antibody (Figure S5C,D). Using orthotopic syngeneic breast cancer models to assess the causal role of NK cells in vivo, we implanted HR^+^ murine cell lines 67NR and EMT6 and performed NK cell depletion with anti‐asialo‐GM1. Anti‐PD‐1 immunotherapy robustly suppressed tumor growth in both models, whereas this therapeutic effect was abrogated upon NK cell depletion (Figure 3H–J and Figure S5E,F). Consistent with the antitumor effects of anti‐PD‐1 therapy, we observed increased abundance of NK cells and CD8^+^ T cells, along with elevated expression of cytotoxic markers following anti‐PD‐1 therapy (Figure 3K,L and Figure S5G). In parallel with the increased infiltration of cytotoxic cells, the expression of key chemokines CCL3, CCL4, and CCL5 was also elevated upon anti‐PD‐1 treatment (Figure S5H). Conversely, the infiltration and cytotoxic marker expression of CD8^+^ T cells, as well as the expression of CCL3, CCL4, and CCL5, were reduced when NK cells were depleted, suggesting that NK cells may contribute to shaping CD8^+^ T cell responses in the HR^+^ murine breast cancer model (Figure 3L and Figure S5H). These findings underscore the cytotoxic and immunomodulatory roles of NK cells in ICI therapy for HR^+^/HER2^−^ breast cancer.

### 2.5 Platinum Induces NK Cell Activation via NF‐κB Pathway

**FIGURE 4:**
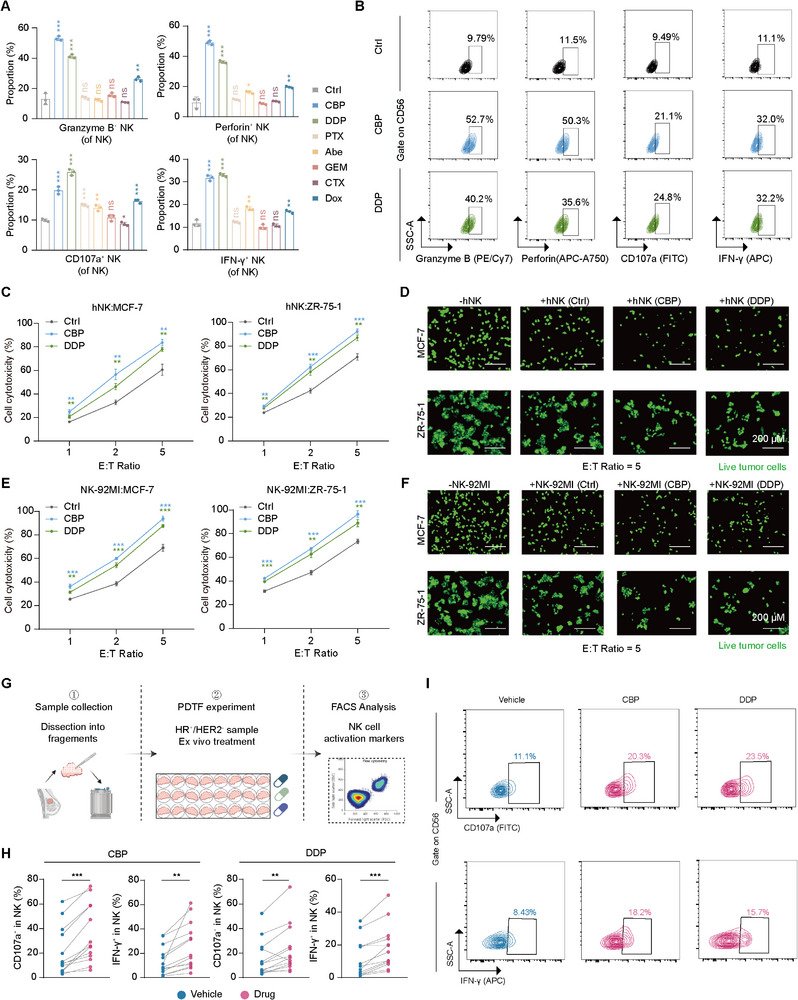
Screening for drugs that activate NK cells in HR^+^/HER2^−^ breast cancer. (A) Statistical graph of flow cytometry representing the expression profile of functional markers in hNK cells treated with various drugs. *n* = 3. A single vehicle control containing the highest final concentration of organic solvent (culture medium with 0.033% DMSO and 0.005% DMF) was included for comparison. Data are presented as mean ± SD. Two‐tailed unpaired Student's *t* test. (B) Representative flow cytometry images representing the expression profile of functional markers in hNK cells treated with various drugs. (C) Cytotoxic effects of hNK cells treated with carboplatin and cisplatin against MCF‐7 and ZR‐75‐1 cells. *n* = 3. Data are presented as mean ± SD. Two‐tailed unpaired Student's *t* test. (D) Representative images representing the cytotoxicity effects of hNK cells treated with cisplatin and carboplatin against MCF‐7 and ZR‐75‐1 cells. E:T ratio = 5. *n* = 3 independent experiments. (E) Cytotoxicity of NK‐92MI cells treated with cisplatin and carboplatin against MCF‐7 and ZR‐75‐1 cells. *n* = 3. Data are presented as mean ± SD. Two‐tailed unpaired Student's *t* test. (F) Representative images representing the cytotoxicity of NK‐92MI cells treated with cisplatin and carboplatin against MCF‐7 and ZR‐75‐1 cells. E:T ratio = 5. *n* = 3 independent experiments. (G) Schematic workflow of the assay for detecting the effect of drugs on NK cells using Patient‐Derived Tumor Fragment (PDTF) models. Flow cytometry acquisition typically yielded approximately 0.5 × 10^5^ to 0.5 × 10^6^ total events per pooled sample. Only samples with at least 1 × 10^4^ viable CD45^+^ events were included for analysis. (H) Statistical graph of flow cytometry representing the expression profile of functional markers in NK cells from PDTF tissues treated with platinum. Data are presented as mean ± SD. Two‐tailed paired Student's *t* test. (I) Representative flow cytometry images representing the expression profile of functional markers in NK cells from PDTF tissues treated with platinum. See also Figure S6. CBP, carboplatin; DDP, cisplatin; PTX, paclitaxel; Abe, Abemaciclib; GEM, gemcitabine; CTX, cyclophosphamide; Dox, doxorubicin.

Given the uncertain efficacy of the NK cell agonist monalizumab in clinical trials [31, 32], we next sought to identify commonly used agents in HR^+^/HER2^−^ breast cancer that could enhance the cytotoxicity of NK cells. In addition to endocrine therapy, CDK4/6 inhibitors and chemotherapy are routinely used systemic treatments in HR^+^/HER2^−^ breast cancer, both in high‐risk early‐stage disease and in advanced settings [33, 34, 35]. We selected representative drugs from major therapeutic classes, including platinum compounds, paclitaxel, anthracyclines, CDK4/6 inhibitors, gemcitabine, and cyclophosphamide, and assessed their effects on NK cell viability in vitro. NK cells were treated with graded concentrations of each agent, and the highest concentration that did not compromise NK cell survival was subsequently used to evaluate NK cell activation (Figure S6A). Compared with other agents, the platinum agents carboplatin and cisplatin strikingly activated NK cells with increased levels of Granzyme B, Perforin, CD107a and IFN‐γ (Figure 4A,B). Consistent with the increased activation markers, platinum‐pretreated NK cells exhibited increased cytotoxicity against HR^+^ breast cancer cells (Figure 4C–F and Figure S6B). We further validated the ability of platinum to activate NK cells using patient‐derived tumor fragment (PDTF) models, which preserve the native tumor microenvironment (Figure 4G). We observed a substantially increased level of NK cell activation in tumors from patients with HR^+^ breast cancer following platinum treatment, whereas other agents showed minimal to no efficacy. (Figure 4H,I and Figure S6C,D).

**FIGURE 5:**
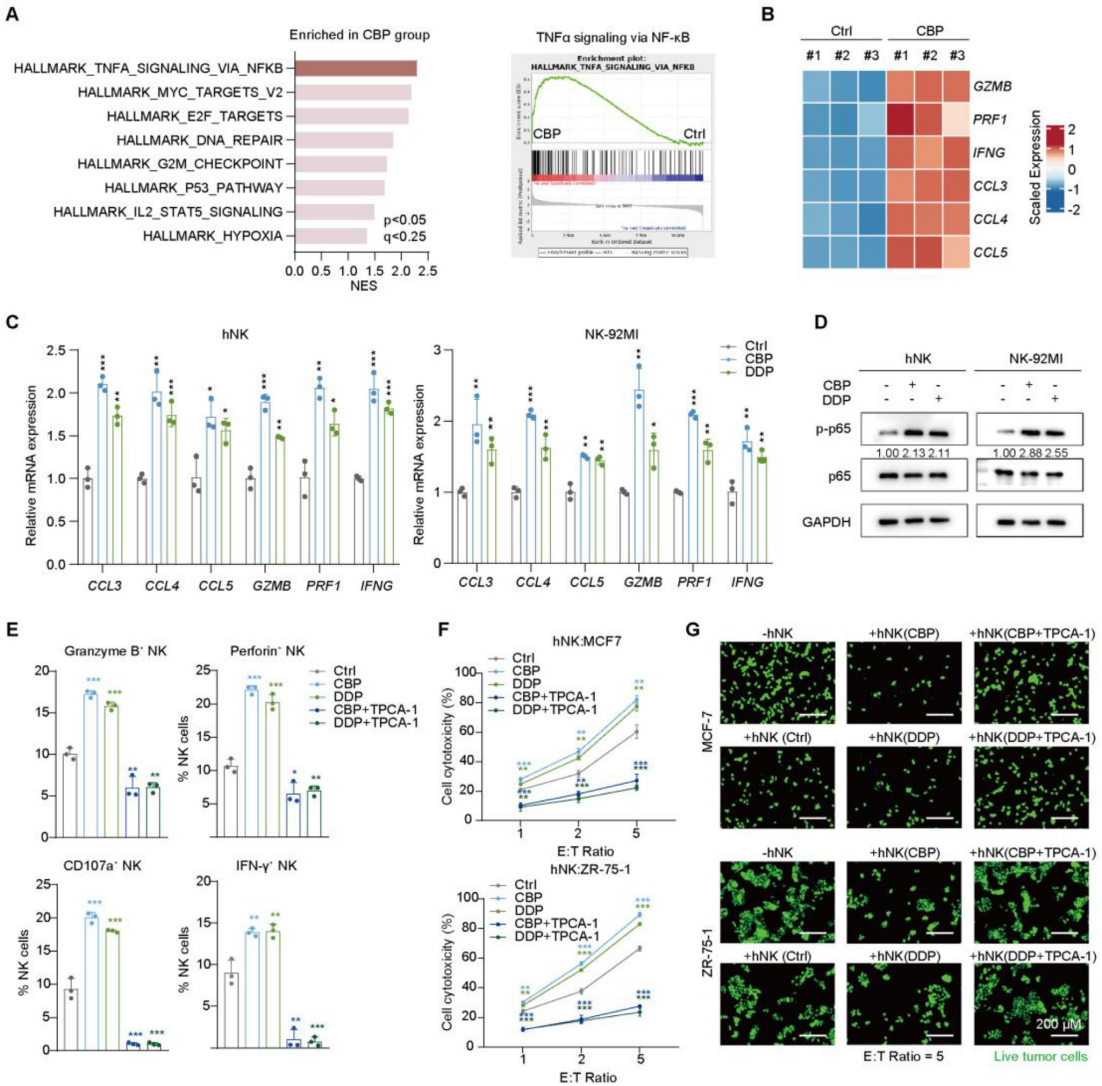
Platinum‐induced NK cell activation via the NF‐κB pathway. (A) RNA‐seq analysis of the enriched pathways in NK‐92MI cells following platinum treatment. (B) RNA‐seq analysis of the expression of NF‐κB pathway downstream target genes in control and carboplatin‐treated NK‐92MI cells. (C) qPCR analysis of the expression of NF‐κB pathway downstream target genes in control and platinum‐treated NK cells. *n* = 3. Data are presented as mean ± SD. Two‐tailed unpaired Student's *t* test. (D) Western blot analysis of the expression of p65 and p‐p65 in control and platinum‐treated NK cells. Densitometric quantification of p‐p65 was performed using ImageJ and normalized to the corresponding control condition, which was set to 1.00. *n* = 3 independent experiments. (E) Flow cytometry analysis of the expression profile of functional markers in hNK cells co‐treated with TPCA‐1 (2 µm) and platinum drugs. Data are presented as mean ± SD. Two‐tailed unpaired Student's *t* test. *n* = 3. (F) Cytotoxicity of hNK cells treated with TPCA‐1 and platinum drugs against MCF‐7 and ZR‐75‐1 cells. *n* = 3. Data are presented as mean ± SD. Two‐tailed unpaired Student's *t* test. (G) Representative images representing the cytotoxicity of hNK cells treated with TPCA‐1 and platinum drugs against MCF‐7 and ZR‐75‐1 cells. E:T ratio = 5. *n* = 3 independent experiments. See also Figure S7. CBP, carboplatin; DDP, cisplatin.

To elucidate the mechanism underlying platinum‐induced NK cell activation, we performed RNA sequencing of NK cells treated with vehicle or carboplatin. Gene set enrichment analysis (GSEA) was performed to investigate altered signaling pathways following carboplatin treatment. The result revealed a marked enrichment of TNFα signaling via the NF‐κB pathway, alongside enrichment of stress‐ and damage‐associated programs, including DNA repair, inflammatory response, and hypoxia signaling (Figure 5A). These results suggest that platinum induces a coordinated cellular stress and inflammatory transcriptional response in NK cells that converges on canonical NF‐κB activation. Consistent with this pathway‐level observation, analysis of the leading‐edge genes driving NF‐κB pathway enrichment revealed a coordinated stress‐responsive transcriptional program, characterized by upregulation of AP‐1 components (e.g., *JUN*, *FOSL2*), NR4A family transcription factors (e.g., *NR4A2*, *NR4A3*), and activation markers and effector genes (e.g., *CD69* and *AREG*) (Figure S7A). We further validated the downstream effectors of this pathway and found that the mRNA expression levels of activation and effector markers (e.g., *GZMB*, *PRF1*, *IFNG*) and chemotactic molecules (e.g., *CCL3*, *CCL4*, *CCL5*) were significantly increased in platinum‐treated NK cells (Figure 5B,C). Consistent with activation of the NF‐κB pathway, platinum treatment was associated with increased phosphorylation of p65 in NK cells (Figure 5D).

To directly interrogate the functional relevance of NF‐κB signaling in platinum‐induced NK cell activation, we pharmacologically perturbed this pathway using TPCA‐1 [36], a selective IKKβ inhibitor that targets the upstream regulatory module of canonical NF‐κB signaling. TPCA‐1 markedly reduced p65 phosphorylation without affecting NK cell viability, as confirmed by CCK‐8 assays and flow cytometric analysis using propidium iodide staining (Figure S7B–E). Importantly, blockade of NF‐κB signaling by TPCA‐1 significantly attenuated the platinum‐induced upregulation of NK cell activation markers and suppressed NK cell‐mediated cytotoxicity against HR^+^/HER2^−^ breast cancer cell lines (Figure 5E–G and Figure S7F). To further exclude compound‐specific or off‐target effects, we performed parallel experiments using BMS‐345541, a structurally distinct IKKβ inhibitor. BMS‐345541 similarly reduced platinum‐induced enhancement of NK cell functional markers and diminished NK cell‐mediated tumor cell killing (Figure S7G,H).

Collectively, these results support a model in which platinum treatment activates canonical NF‐κB signaling in NK cells, thereby promoting NK cell activation and cytotoxic function.

### 2.6 Platinum Enhances Immunotherapy Responsiveness Through Activating NK Cells

**FIGURE 6:**
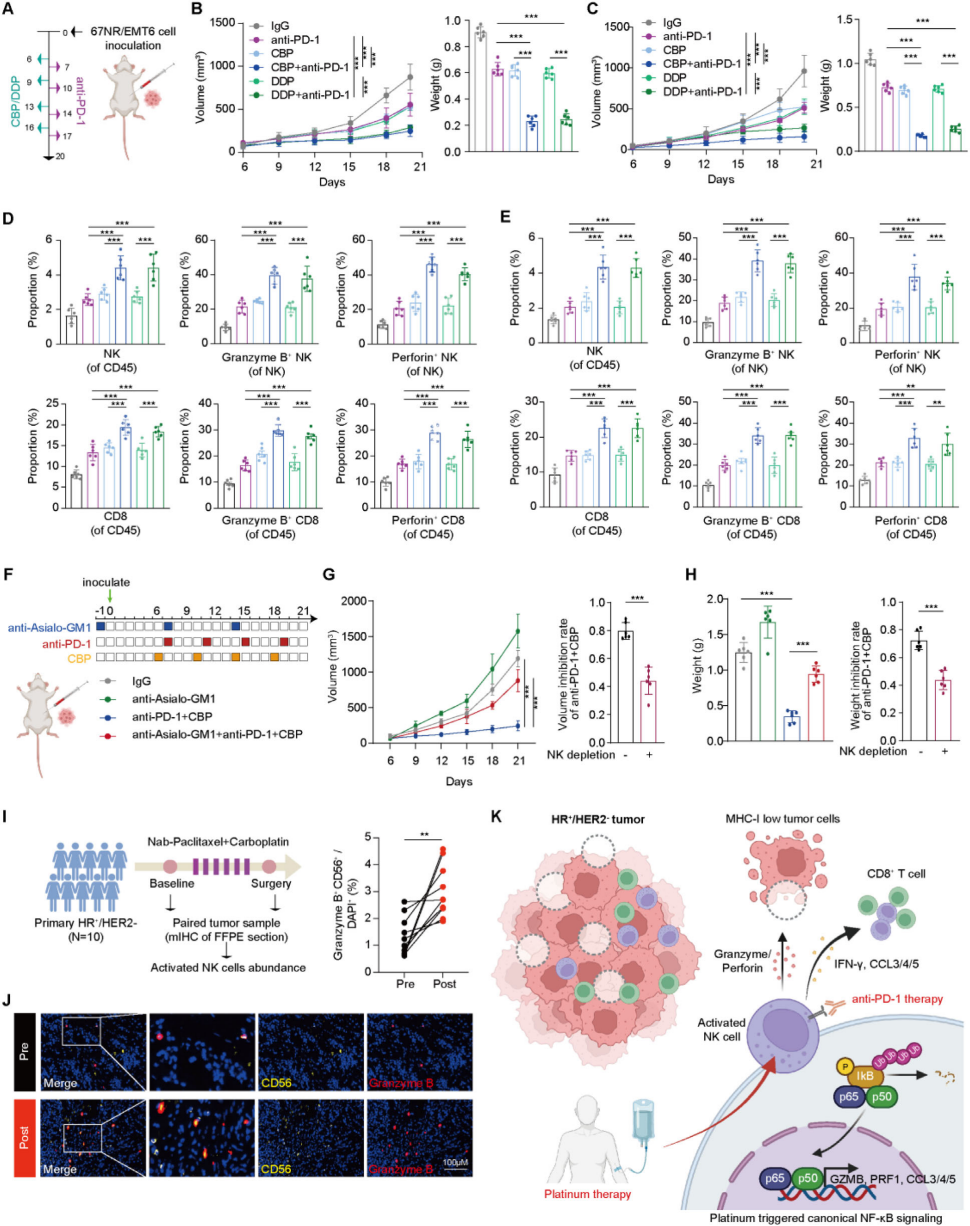
Synergistic effect of platinum and immunotherapy in HR^+^/HER2^−^ breast cancer. (A) Overview of the orthotopic isograft models for combination therapy. (B) Growth curves and tumor weights at the endpoint in mice treated with anti‐PD‐1 antibody and platinum in the 67NR orthotopic tumor model (*n* = 6 per group). The data are presented as mean ± SD, and the data are compared using two‐way ANOVA for growth curves and Student's *t* test for tumor weights. (C) Growth curves and tumor weights at the endpoint in mice treated with anti‐PD‐1 antibody and platinum in the EMT6 orthotopic tumor model (*n* = 6 per group). The data are presented as mean ± SD, and the data are compared using two‐way ANOVA for growth curves and Student's *t* test for tumor weights. (D) Infiltration and functional markers of NK cells and CD8^+^ *T* cells measured by flow cytometry from 67NR tumors. *n* = 6. Data are presented as mean ± SD. Two‐tailed unpaired Student's *t* test. (E) Infiltration and functional markers of NK cells and CD8^+^ *T* cells measured by flow cytometry from tumors in EMT6 tumor. *n* = 6. Data are presented as mean ± SD. Two‐tailed unpaired Student's *t* test. (F) Schematic overview of the EMT6 orthotopic isograft model and treatment schedule for anti‐PD‐1 plus carboplatin therapy with or without NK cell depletion. (G) Tumor growth curves (left) and tumor volume inhibition rates (right) of anti‐PD‐1 plus carboplatin therapy in the presence or absence of NK cell depletion. Data are presented as mean ± SD. Two‐sided two‐way ANOVA for growth curves and two‐sided Student's *t* test for tumor volume comparison at the endpoint. (H) Tumor weights (left) and tumor weight inhibition rates (right) of anti‐PD‐1 plus carboplatin therapy in the presence or absence of NK cell depletion. Data are presented as mean ± SD. Two‐tailed unpaired Student's *t* test. (I) Immunofluorescence analysis of Granzyme B^+^ CD56^+^ NK cells in paired tumor sections from a platinum‐based chemotherapy cohort. Data are presented as mean ± SD. Two‐tailed paired Student's *t* test. (J) Representative multiplex immunohistochemistry images of Granzyme B^+^ CD56^+^ NK cells in paired tumor sections from a platinum‐based chemotherapy cohort. (K) A schematic highlighting the major findings of this study. See also Figure S8. CBP, carboplatin; DDP, cisplatin.

To further validate whether platinum could enhance immunotherapy, we conducted combination therapy using orthotopic isograft models. We established orthotopic isograft models utilizing the HR^+^ murine breast cancer cell lines 67NR and EMT6. The tumor‐bearing mice were then treated with carboplatin or cisplatin in combination with an anti‐PD‐1 antibody (Figure 6A). Remarkably, the carboplatin‐ or cisplatin‐treated groups showed significantly increased anti‐tumor responses when combined with anti‐PD‐1 immunotherapy, as evidenced by the pronounced inhibition of tumor growth (Figure 6B,C). Flow cytometry analysis of tumor tissues suggested that both ICI and platinum could activate NK cells within the tumor microenvironment (Figure 6D,E and Figure S8A,B). The combination of these two agents further enhanced the activation level of NK cells, accompanied by the infiltration and activation of CD8^+^ T cells, suggesting an immune‐activated microenvironment (Figures 6D,E and Figure S8A–D). To directly determine whether NK cells are required for the antitumor efficacy of anti‐PD‐1 plus platinum therapy, we next performed NK cell depletion in the combination treatment model (Figure 6F). Consistent with the above results, anti‐PD‐1 and carboplatin combination therapy robustly suppressed tumor growth compared with control groups. Notably, depletion of NK cells markedly weakened the antitumor effect of the combination therapy (Figure 6G,H and Figure S8E). Together, these findings provide functional evidence that NK cells are required for platinum‐mediated enhancement of immunotherapy responsiveness.

### 2.7 Clinical Cohort Validation of the Efficacy of Platinum on NK Cells

Our previous in vitro and in vivo studies demonstrated that platinum agents can activate NK cells. Leveraging these insights, we used specimens from a clinical cohort to further validate the effect of platinum drugs on NK cell activity. We collected paired biopsy samples from ten patients before and after they underwent a platinum regimen and conducted mIHC staining to evaluate the expression levels of CD56 and Granzyme B. The results revealed a significant increase in the proportion of activated NK cells in the post‐treatment tumor microenvironment compared to the pre‐treatment samples (Figure 6I,J). These findings confirm that platinum treatment enhances NK cell activation in the clinical setting, supporting the potential of platinum‐based therapies to improve immunotherapy outcomes in HR^+^/HER2^−^ breast cancer.

## 3 Discussion

The efficacy of immunotherapy for HR^+^/HER2^−^ breast cancer remains limited, underscoring the need to better characterize its tumor microenvironment to identify responsive populations. Our study contributes to this effort by exploring several key aspects. First, our findings suggest the existence of three distinct microenvironment phenotypes within HR^+^/HER2^−^ breast cancer, including an “immune hot” subgroup characterized by activated immune cells, particularly cytotoxic lymphocytes. Second, we identified an association between the abundance of activated NK cells and a favorable response to anti‐PD‐(L)1 therapy, indicating their potential as predictive biomarkers. Finally, our in vitro data led us to hypothesize that platinum‐based chemotherapy may enhance anti‐PD‐(L)1 efficacy by directly activating NK cells through the NF‐κB signaling pathway (Figure 6K).

Previous studies have identified predictive biomarkers for immunotherapy response in HR^+^/HER2^−^ breast cancer. For instance, the GIADA trial utilized stromal TIL scores and gene expression data to investigate features associated with pCR to neoadjuvant ICI combined with chemotherapy, revealing that a combined score of the basal subtype and TILs could effectively predict pCR [19]. In translational research of the I‐SPY2 trial, deconvolution analysis of gene expression data indicated that B cell scores were associated with sensitivity, whereas mast cell scores were linked to resistance to neoadjuvant ICI combined with chemotherapy [29]. Although these studies have shed light on predictive biomarkers for immunotherapy efficacy in HR^+^/HER2^−^ breast cancer, their clinical applications have been limited. Here, we employed high‐resolution single‐cell transcriptomic technology to comprehensively explore predictive biomarkers of anti‐PD‐(L)1 efficacy in HR^+^/HER2^−^ breast cancer. We prospectively collected baseline tumor specimens from patients with HR^+^/HER2^−^ breast cancer undergoing anti‐PD‐(L)1 immunotherapy and performed scRNA‐seq. By comparing the cellular composition of the tumor microenvironment between the different treatment response groups, we found that tumors from patients with better treatment outcomes were significantly enriched in activated NK cells, which exhibited enhanced cytotoxicity and immunoregulatory functions. This finding was consistent with observations in external cohorts of HR^+^/HER2^−^ patients receiving neoadjuvant anti‐PD‐(L)1 therapy.

NK cells are vital components of the innate immune system, primarily functioning by recognizing a balance of inhibitory and activating signals on the surface of target cells. They can effectively attack tumor cells that evade CD8^+^ T cell attacks owing to the absence of MHC class I molecules [37]. Beyond their direct cytotoxic activity, NK cells also play important regulatory roles in shaping adaptive immune responses. Specifically, NK cells secrete cytokines such as IFN‐γ and TNF‐α that modulate the function of adaptive immune cells [38, 39]. In addition, NK cell‐derived chemokines, including *CCL3, CCL4, CCL5*, and *XCL1*, facilitate the recruitment and activation of dendritic cells and other hematopoietic cells within the tumor microenvironment [40]. Moreover, NK cell‐mediated killing of tumor or infected cells can enhance antigen cross‐presentation to CD8^+^T cells, thereby amplifying adaptive immunity [41]. Collectively, these regulatory and effector functions position NK cells as a critical bridge between innate and adaptive immune responses. Importantly, the function of NK cell is tightly regulated within the tumor microenvironment. Studies have shown that checkpoint molecules such as PD‐1 and PD‐L1 are present on NK cells, and their function can be suppressed through the PD‐1/PD‐L1 axis. Relieving this suppression could bolster the anti‐tumor capabilities of NK cells [42]. Consistent with this concept, preclinical studies have highlighted a critical contribution of NK cells to the efficacy of PD‐1/PD‐L1 blockade in melanoma, colon cancer, and prostate cancer models [43]. In breast cancer, however, mechanistic studies of NK cells have predominantly focused on TNBC. Prior work identified an immature NK cell subset within TNBC tumors that promotes tumor progression by secreting Wnt ligands that enhance cancer stemness and support a pro‐tumoral microenvironment [28]. In addition, intratumoral heterogeneity of MHC class I expression in TNBC has been shown to impair responses to ICIs, while simultaneously increasing NK cell infiltration. In such settings, therapeutic blockade of NK inhibitory receptors can overcome resistance to PD‐L1‐based therapies in MHC‐I heterogeneous tumors [44]. By contrast, the role of NK cells in modulating immunotherapy responses in HR^+^/HER2^−^ breast cancer remains poorly defined. HR^+^/HER2^−^ tumors are typically characterized by limited immune infiltration and reduced expression of MHC class I molecules and immune checkpoint targets, which may constrain effective T cell‐dominant antitumor immunity [17, 22]. In this context, NK cells‐capable of responding to “missing‐self” signals‐may represent a more critical cytotoxic effector population for HR^+^/HER2^−^ breast cancers. In the current study, we utilized single‐cell transcriptomic data from a prospective immunotherapy cohort to identify activated NK cells as potential biomarkers for the enhanced efficacy of anti‐PD‐(L)1 therapy. By contrast, resting NK cells, which encompassed the immature NK cell subset described by Thacker et al. [44], showed no association between their abundance and immunotherapy response. Mechanistically, our analysis suggests that anti‐PD‐(L)1 treatment may bolster the cytotoxicity and immunoregulatory function of NK cells, enabling them to effectively target HR^+^/HER2^−^ tumors. This observation provides additional insights into the relationship between NK cells and breast cancer immunotherapy.

From a therapeutic perspective, NK cell‐based strategies can be broadly categorized into several approaches, including adoptive NK cell transfer, cytokine‐based NK cell stimulation, exploitation of antibody‐dependent cellular cytotoxicity (ADCC), and targeting of NK cell immune checkpoints. However, despite substantial preclinical promise, major challenges remain, including limited in vivo persistence and expansion of NK cells, functional suppression by the immunosuppressive tumor microenvironment, and inefficient tumor infiltration, particularly in solid tumors [45]. Among NK‐targeted therapies, inhibitory receptor blockade has shown initial promise. For example, the NKG2A inhibitor monalizumab demonstrated encouraging activity in early‐phase studies [46, 47]. However, the Phase III INTERLINK‐1 trial, which combined monalizumab with cetuximab, did not enhance overall survival in patients with recurrent and metastatic head and neck squamous cell carcinoma, underscoring the difficulty of translating NK‐targeted strategies into durable clinical benefit [31]. In the absence of clinically approved NK‐specific therapies, increasing attention has turned toward whether conventional anticancer treatments can be repurposed to indirectly or directly modulate NK cell function. Previous studies have shown that certain chemotherapeutic and targeted regimens can augment immunotherapy efficacy through tumor‐centric mechanisms, including induction of immunogenic cell death [48], upregulation of MHC molecules, and activation of immune‐related pathways in tumor cells [49]. In addition, prior studies have predominantly focused on the direct immunomodulatory effects of chemotherapy on immune cells, particularly cytotoxic *T* cells and regulatory *T* cells [50, 51, 52]. However, the direct effects of these agents on NK cells remain incompletely understood. Consistent with this notion, clinical and preclinical studies, such as the TONIC trial and ovarian cancer mouse models, have reported increased expression of cytotoxicity‐associated genes or enhanced NK cell infiltration following platinum‐based chemotherapy [53, 54]. Notably, these observations primarily describe correlative changes at the tissue or transcriptional level, without addressing whether platinum agents can intrinsically activate NK cells. Mechanistic investigations have largely focused on chemotherapy‐induced modulation of NK cell ligands on tumor cells [55, 56], leaving direct drug‐NK cell interactions insufficiently characterized. In contrast, our study provides direct mechanistic evidence that platinum chemotherapy acts as an immune cell‐intrinsic activator of NK cells through activation of the NF‐κB signaling pathway. This finding establishes a clear conceptual distinction from prior studies and provides a mechanistic rationale for leveraging platinum‐induced NK activation to enhance responsiveness to immune checkpoint blockade in HR^+^/HER2^−^ breast cancer.

In parallel, we observed enrichment of endothelial cells in non‐responders within the HR^+^/HER2^−^ scRNA cohort. Tumor endothelial cells are increasingly recognized as active regulators of immune exclusion, limiting immune cell trafficking and promoting immunosuppressive tumor microenvironments [57, 58]. Aberrant endothelial cells can impede immune infiltration through disorganized vasculature, reduced expression of adhesion molecules, and altered chemokine gradients, while also directly suppressing immune effector functions via expression of immune checkpoint ligands and secretion of inhibitory factors [59, 60, 61, 62]. In this context, enrichment of endothelial cells in non‐responders likely reflects a vascular and stromal state that further exacerbates immune exclusion, whereas reduced endothelial cells in responders may indicate a more permissive microenvironment that facilitates immune cell access and supports immunotherapy efficacy. Together, these findings suggest that modulation of tumor endothelial cells, including anti‐angiogenic strategies, may represent a complementary approach to improve immunotherapy responsiveness in HR^+^/HER2^−^ breast cancer.

Our study has several limitations. First, the scRNA analysis was performed on a limited number of samples, which constrains the statistical robustness of our findings. While incorporating the external I‐SPY2 cohort provided supportive evidence, the predictive value of activated NK cells must be validated in larger, real‐world cohorts alongside the development of clinically applicable detection methods. Second, in vivo NK cell depletion in this study relied on anti‐asialo‐GM1 in BALB/c‐based HR^+^/HER2^−^ murine models. Although this approach is widely used, it is not strictly NK‐specific and may exert limited off‐target effects on other immune subsets. While we observed robust NK cell depletion with minimal impact on myeloid compartments, this methodological limitation should be considered when interpreting the in vivo depletion experiments. Third, it is important to recognize that the therapeutic efficacy of NK cell‐based approaches can be constrained by inefficient tumor infiltration and immunosuppressive tumor microenvironments in solid tumors [45, 63]. Accordingly, although platinum treatment enhances NK cell cytotoxic and immunoregulatory functions, this strategy alone may be insufficient to overcome quantitative and spatial limitations on NK cells within tumors. Future studies should therefore explore rational combination strategies that integrate platinum‐based chemotherapy with NK cell‐directed approaches, including immune checkpoint blockade targeting NK inhibitory receptors (e.g., NKG2A and TIGIT), multispecific NK cell engagers, or adoptive NK cell therapies [39, 64, 65, 66]. Finally, future studies should explore pharmacologic agents with NK cell‐activating potential beyond standard breast cancer therapies, particularly those that enhance NK cell metabolic fitness [67, 68, 69, 70]. Targeting metabolic checkpoints may represent promising directions to amplify platinum‐induced NK cell activation and improve immunotherapy efficacy.

In conclusion, our study characterized the microenvironmental heterogeneity of HR^+^/HER2^−^ breast cancer and revealed a significant association between the abundance of activated NK cells and favorable immunotherapy outcomes. Our findings suggest that activated NK cells represent a promising biomarker for anti‐PD‐(L)1 sensitivity, and that their anti‐tumor functions may be potentiated by anti‐PD‐(L)1 therapy. Furthermore, we proposed a novel mechanism by which platinum drugs augment anti‐PD‐(L)1 efficacy through direct NK cell activation. These findings warrant further investigation to establish their clinical utility and confirm the proposed mechanisms in patients. Collectively, our work provides a strong rationale for future studies focusing on NK cells to develop more effective immunotherapeutic strategies for HR^+^/HER2^−^ breast cancer.

## 4 Methods

### 4.1 FUSCC Multi‐Omics Cohort

The previously established Chinese breast cancer cohort from the Fudan University Cancer Center (FUSCC) included 913 treatment‐naïve female patients who underwent surgery at the Department of Breast Surgery at FUSCC [17, 22]. The status of ER, PR, and HER2 was confirmed by two experienced pathologists using immunohistochemical analysis and in situ hybridization. A cutoff of <1% positively stained cells was used to define ER/PR negativity. Clinicopathological features, including age, histological tumor type, tumor size, axillary lymph node status, grade, adjuvant therapies, and ER, PR, HER2, and Ki67 statuses, were collected. The primary focus of this study was luminal‐type breast cancer (HR^+^/HER2^−^), defined as tumors that were HR‐positive (ER+ or PR+) with a HER2 status of 0 or 1+ by immunohistochemistry or those with a HER2 status of 2+ by immunohistochemistry but confirmed negative by FISH. For comparative purposes, patients with Triple‐Negative Breast Cancer (TNBC), a subtype extensively studied in prior microenvironmental research, were also included. This combination formed the final FUSCC HER2^−^negative cohort comprising a total of 672 patients. Multi‐omics data were available for this cohort, including transcriptome sequencing for all 672 patients, WES of matched tumor‐blood pairs for 576 patients, and CNA data from tumor tissue for 480 patients.

### 4.2 FUSCC Immunotherapy Cohorts

#### 4.2.1 scRNA‐Seq Discovery Cohort

This cohort included 11 female patients with HR^+^/HER2^−^ breast cancer who received neoadjuvant anti‐PD‐L1 immunotherapy at FUSCC. The detailed clinicopathological data are presented in Table S1. Fresh tumor biopsies were prospectively collected at baseline for single‐cell RNA sequencing and parallel paraffin embedding. Following the completion of neoadjuvant therapy and surgery, patients were classified by pathological assessment using the residual cancer burden (RCB) index as either responders (R; RCB‐I/II) or non‐responders (NR; RCB‐III).

#### 4.2.2 Validation Cohort

This cohort consisted of 24 female patients with HR^+^/HER2^−^ breast cancer who received neoadjuvant anti‐PD‐L1 immunotherapy at FUSCC. Pre‐treatment formalin‐fixed paraffin‐embedded (FFPE) biopsies were used for multiplex immunohistochemistry (mIHC) staining. The detailed clinicopathological data are presented in Table S2.

The study was approved by the FUSCC Ethics Committee, and all participants provided written informed consent before enrollment.

### 4.3 I‐SPY2 Immunotherapy Cohort

Data from the I‐SPY2 trial (NCT01042379) were used to evaluate the predictive potential of NK cells in the response to anti‐PD‐(L)1 immunotherapy. We analyzed publicly available transcriptome and outcome data from two ICI therapy arms: an anti‐PD‐1 arm (pembrolizumab/paclitaxel; *n *= 40 HR^+^/HER2^−^ and 29 TNBC patients) and an anti‐PD‐L1 arm (durvalumab/olaparib/paclitaxel; *n* = 50 HR^+^/HER2^−^ and 21 TNBC patients) [20, 21, 29].

### 4.4 Bioinformatics Analysis

#### 4.4.1 Transcriptome‐Based Microenvironment Subtyping

The reference microenvironment compendium, utilized for calculating microenvironment cell abundance, comprises 364 genes representing 24 distinct cell subsets, including CIBERSORT [71] (22 immune cells) and MCP‐counter [72] (fibroblasts and endothelial cells). We then applied the single‐sample gene set enrichment analysis (ssGSEA) function in the R package ‘GSVA’ to calculate the score of each microenvironment cell type, and identified groups of samples with similar microenvironmental characteristics through k‐means (‘kmeans’ function in R) analysis. This assessment was conducted in the TCGA cohort. For validation, the abundance of microenvironmental cell types was estimated using CIBERSORT [71] with default settings and xCell [73].

#### 4.4.2 Cancer‐Immunity Cycle Analysis Method

Based on the classic literature on the cancer‐immunity cycle [74], the immune system undergoes a series of key stages in combating tumors: (1) Release of antigens, (2) Presentation of antigens, (3) Initiation and activation of *T* cells, (4) Chemotaxis of *T* cells, (5) Infiltration of *T* cells, (6) Recognition by *T* cells, (7) Killing by *T* cells. In this cycle, the initiation and activation of *T* cells mainly occur in the regional lymph nodes. To gain a deeper understanding of the characteristics of the core components of the cancer‐immunity cycle in different tumor samples, we selected six of these stages, excluding the initiation and activation of *T* cells, for detailed analysis. The genes included in each step are presented in Table S3.

#### 4.4.3 Tumor Ecotype Analysis Method

EcoTyper is a machine‐learning‐based analytical tool capable of identifying and validating cellular states and ecotypes from bulk, single‐cell, and spatial transcriptome data [24]. Utilizing transcriptome data, we executed the EcoTyper_recovery_bulk.R script in alignment with the EcoTyper analysis pipeline to ascertain the ecotype characteristics of tumor samples within our cohort.

#### 4.4.4 Signature Assessment

The endocrine therapy sensitivity score was calculated according to a previous study [75]. The calculation of NK resistant and sensitive signatures was performed by the ‘gsva’ function in the R package ‘GSVA’ using a set of related genes [30].

### 4.5 scRNA‐Seq Data Analysis

#### 4.5.1 Data Processing

Cell Ranger (v3.1.0) was used to process cellular barcodes, align reads to the reference genome using STAR, and generate a gene‐barcode count matrix for each sample. The unique molecular identifier (UMI) count matrix was further analyzed using Seurat (v4.0.5) in R. To identify and exclude doublets and empty droplets, the R packages scDblFinder (v1.6.0) and DropletUtils (v1.12.3) were used. We filtered out low‐quality cells with UMI/gene numbers below 200 or above 8000 and further discarded those with > 20% mitochondrial gene counts. After applying these QC criteria, a total of 69784 single cells remained for further downstream analyses.

#### 4.5.2 Cell Clustering, Visualization, and Annotation

To mitigate batch effects from the samples, we employed harmony (v0.1.0) to align data across batches. After batch correction, the integrated matrix was processed in Seurat following the standard workflow. We utilized the top 50 principal components for Louvain clustering at a resolution of 0.6 and applied uniform manifold approximation and projection (UMAP) for dimensionality reduction and visualization. To annotate cell clusters, SingleR (v1.6.1) was used to independently deduce cell origins against the ‘Human Primary Cell Atlas’ reference dataset [76].

#### 4.5.3 Single‐Cell Copy Number Calling and Cancer Cell Identification

We utilized the R package “inferCNV” (v1.8.1) with optimized parameters to determine copy number variations in individual cells. Immune cells served as a reference to establish the baseline copy number profiles against which other cells were assessed. To distinguish neoplastic cells from epithelial cells, all epithelial and reference cells involved in inferCNV analysis were categorized into 15 clusters using k‐means clustering in R based on their genomic locus copy number profiles. Epithelial cells that clustered closely with reference cells exhibiting minimal CNV changes were classified as normal, whereas those forming distinct clusters with a higher number of CNV alterations were identified as tumor cells.

#### 4.5.4 STARTRAC Analysis

STARTRAC (Single *T* cell Analysis by RNA sequencing and TCR tracking) is a quantitative framework originally developed to quantitatively describe tissue distribution, clonal expansion, migration, and developmental transition or differentiation of *T* cells based on single‐cell transcriptomic data [25, 26]. STARTRAC analysis was performed as a complementary approach to direct frequency‐based comparisons to assess the distributional preference of cell clusters across clinical response groups. The function ‘calTissueDist’ in R package ‘Startrac’ was used.

### 4.6 Cell Lines

The human breast cancer cell lines (MCF‐7, T47D, ZR‐75‐1, MDA‐MB‐436, MDA‐MB‐157, MDA‐MB‐468, MDA‐MB‐231, and SUM159) and murine breast cancer cell lines (67NR and EMT6) were cultured in DMEM (Dulbecco's Modified Eagle Medium), supplemented with 10% fetal bovine serum (FBS) and 1% penicillin–streptomycin. The BT‐549 cell line was cultured in RPMI‐1640 medium with 10% FBS, 1% penicillin‐streptomycin, and 0.023 U/mL insulin. The HCC1937 cell line was cultured in RPMI‐1640 medium with 10% FBS and 1% penicillin‐streptomycin. NK‐92MI cells were cultured in MEM α medium supplemented with 12.5% horse serum, 12.5% FBS, 1% penicillin–streptomycin (P/S), 0.2 mm inositol, 0.1 mm β‐mercaptoethanol, and 0.02 mm folic acid. Human peripheral blood‐derived NK cells (hNK) were isolated from PBMCs using Ficoll density gradient centrifugation, and expanded using the OptiVitro NK Cell Expansion Kit (ExCell). The NK cells were cultured in OptiVitro NK Cell Expansion Medium, prepared by supplementing OptiVitro NK Cell Serum‐Free Base Medium with OptiVitro Immune Cell Additive and cytokines (OptiVitro Cytokine I, II, and III) according to the manufacturer's instructions. All cell lines were maintained in a humidified incubator at 37°C with 5% CO2. All cell lines were confirmed to be mycoplasma‐negative and cultured according to the guidelines.

### 4.7 Isolation of Human Primary NK Cells

Peripheral blood mononuclear cells (PBMCs) were isolated from human peripheral blood using Ficoll (17144002, Cytiva) density gradient centrifugation. Primary human NK (hNK) cells were subsequently expanded from isolated PBMCs using the OptiVitro NK Cell Expansion Serum‐free Kit P01 (NE000‐N022, ExCell, China), according to the manufacturer's instructions. This kit provides a feeder‐free culture system designed for robust expansion of functional NK cells. The system included a basal medium and a supplement containing a cytokine cocktail primarily composed of recombinant human Interleukin‐2 (IL‐2) and Interleukin‐15 (IL‐15) to drive NK cell proliferation and activation.

### 4.8 NK Cell‐Mediated Cytotoxicity Assay

Target and NK cells were seeded at effector‐to‐target ratios of 1:1, 2:1, and 5:1 in 384‐well plates. After 4 h of co‐culture, the CellTiter‐Glo cell viability assay (Promega) was performed according to the manufacturer's protocol. Cytotoxicity was calculated as follows: (1 ‐ (co‐culture well signal—NK cell control well signal)/tumor cell control well signal) × 100%. For the acquisition of fluorescence images, target cells and NK cells were seeded at an effector‐to‐target ratio of 5:1 in 96‐well plates and co‐cultured for 4 h. The NK cells were washed away, and the live target cells were stained with calcein‐AM (Solarbio) and visualized using a fluorescence microscope. For flow cytometry‐based quantification of tumor cell death, tumor cells were pre‐labeled with CellTrace Deep Red (CTDR) dye (maokangbio, #MX4110) and subsequently co‐cultured with NK cells at an effector‐to‐target ratio of 5:1 for 4 h. Following co‐culture, tumor cell death was quantified by flow cytometry using CTDR to identify tumor cells and Zombie Dye to detect dead cells. For platinum pretreatment assays, NK cells were incubated with carboplatin or cisplatin for 48 h, followed by extensive washing to remove residual drug before co‐culture with target cells.

### 4.9 Immunohistochemical Staining

Paraffin‐embedded tissue sections were first heated at 60°C for 4 h to remove paraffin, followed by treatment with xylene and a series of graded alcohols. To expose the antigens, slides were boiled in a solution of citrate or EDTA for antigen retrieval. After cooling, the slides were treated with blocking solution consisting of 2% goat serum, 2% bovine serum albumin, and 0.05% Tween 20 in PBS for 10 min at room temperature. They were then incubated with primary antibodies overnight at 4°C. The sections were subsequently covered with a horseradish peroxidase (HRP)‐linked secondary antibody (GeneTech) for 30 min at room temperature and developed using a 3,3′‐diaminobenzidine substrate (GeneTech). Slides were counterstained with hematoxylin, dehydrated using a series of alcohol solutions, and mounted with coverslips and mounting medium. The density of positive cells was assessed by counting under a high‐power field of view. For details on antibody use, please refer to Table S4.

### 4.10 Multiplex Immunohistochemistry Assay (mIHC)

Multiplex immunohistochemistry (CD56 and Granzyme B) was performed on FFPE sections from the FUSCC cohort as previously described, according to the protocol of the Akoya 7‐color Polaris kit (Akoya). Briefly, FFPE sections were deparaffinized, and antigen retrieval was performed. Following the blocking step, the slides were incubated with primary antibodies, and the signal was amplified using opal fluorophores, followed by antibody removal. Slides were counterstained with spectral DAPI. Sequential imaging was performed for each channel, and positivity thresholds were established for each channel using Vectra Polaris software. The resulting data were utilized to quantify the cellular phenotypes of interest using QuPath software. For details on antibody use, please refer to Table S4.

### 4.11 Flow Cytometry

For fresh tissues from mouse models, tissues were digested with Dispase I, Dispase II, and hyaluronidase for 60 min at 37°C. The digested tissues were then filtered through a 70 µm cell strainer and subjected to red blood cell lysis. A total of 1 × 10^6^ cells per sample were used for flow cytometry analysis, according to the manufacturer's protocol (https://www.biolegend.com/protocols/cell‐surfaceflow‐cytometry‐staining‐protocol/4283/). Briefly, the cells were stained for viability with Zombie Dye and blocked with CD16/32 before staining with antibody panels. Surface staining was performed using 5 µL of surface dye in staining buffer at 4°C in the dark for 30 min, followed by centrifugation and a wash. Cells were fixed with a fixative at room temperature in the dark for 30 min, centrifuged, and washed twice with fixative/wash buffer. Intracellular staining was performed using 5 µL of intracellular dye in fixation buffer at 4°C in the dark for 30 min, followed by centrifugation, a wash, and resuspension in staining buffer. The samples were analyzed within 4 h using a flow cytometer. Flow cytometry data were generated on a Beckman Cytomics FC 500 BD FACSCanto II and analyzed using the FlowJo v10 software. For details on antibody use, please refer to Table S4.

### 4.12 RNA Preparation and Real‐Time Quantitative Reverse Transcription (RT‐qPCR)

RNA was isolated from NK cells using the RNeasy Mini Kit (Qiagen) according to the manufacturer's instructions. The purified RNA was then converted into cDNA using the HiScript III RT SuperMix for qPCR (Vazyme). RT‐qPCR analysis was conducted using the ChamQ SYBR Color qPCR Master Mix (Vazyme) on a QuantStudio 6 Flex Real‐Time PCR System (Applied Biosystems). Gene expression levels were quantified using the 2^−ΔΔCt^ method with β‐actin as the reference gene for normalization. The primer sequences used for RT‐qPCR are listed in Table S5.

### 4.13 RNA‐Seq and GSEA Analysis

A total of 1 µg of RNA samples was processed with VAHTS mRNA capture beads (Vazyme, China) to selectively enrich polyA+ RNAs before constructing RNA‐seq libraries. The VAHTS mRNA‐seq v2 library preparation kit compatible with the Illumina Xten system (Vazyme, Nanjing, China) was utilized to generate the RNA‐seq libraries following the manufacturer's protocol. In summary, approximately 100 ng of polyA+ RNA was fragmented and reverse transcribed into double‐stranded cDNA. These cDNA fragments underwent end repair, adenylation of 3' ends, and ligation of sequencing adaptors. The resulting products were purified and subjected to 12 cycles of PCR amplification to produce the final cDNA libraries. The libraries were then sequenced using a 150 bp paired‐end Illumina sequencing run. The sequencing data were aligned to the human genome GRCh38 using HISAT2. Gene expression levels were quantified as fragments per kilobase of transcript per million mapped reads (FPKM). Gene set enrichment analysis (GSEA) was performed using GSEA software (v4.3.2) and the molecular signature database (v7.4).

### 4.14 Western Blotting

Cellular proteins were extracted using an SDS lysis buffer composed of 50 mm Tris (pH 8.1), 1 mm EDTA, 1% SDS, 1 mm fresh dithiothreitol, sodium fluoride, and leupeptin. Protein concentrations were measured using a BCA Protein Assay Kit (Solarbio, Beijing, China). Subsequently, 20 µg of protein was resolved by SDS‐PAGE and transferred to polyvinylidene difluoride membranes (Millipore). The membranes were incubated with the respective primary antibodies, followed by incubation with HRP‐conjugated secondary antibodies, and signals were detected using enhanced chemiluminescence. For details on antibody use, please refer to Table S4.

### 4.15 Chemicals

TPCA‐1 (2 µm; Selleck Chemicals, #S2824) and BMS‐345541 (4 µm; Selleck Chemicals, #S8044) were used for NF‐kB pathway inhibition. Paclitaxel (#S1150), doxorubicin (#E2516), Abemaciclib (#S5716), and gemcitabine (#S1714) were purchased from Selleck Chemicals. Cisplatin (#HY‐17394) and cyclophosphamide (#HY‐17420) were purchased from MedChem. Express. Carboplatin (#C2538) was purchased from Sigma–Aldrich. For all experiments, vehicle controls consisting of the corresponding solvents were included at equivalent volumes. For experiments involving multiple agents, the final concentrations of organic solvents in the culture medium were carefully controlled and kept at or below 0.1%. Under these conditions, a single vehicle control containing the highest final concentration of solvent used across all treatment groups was included for comparison. The solvents used for the preparation of all chemotherapeutic agents are summarized in Table S6.

### 4.16 Patient‐Derived Organoids

Patient‐derived organoid models were established as previously described to preserve the epithelial characteristics of breast tumors [77]. These models were used to assess tumor cell‐intrinsic susceptibility to NK cell‐mediated cytotoxicity. The organoids used in this study were obtained from postsurgical samples of HR^+^/HER2^−^ and TNBC patients who underwent breast surgery at the Department of Breast Surgery, FUSCC. The organoids were cultured according to established protocols [77, 78, 79], suspended in Basement Membrane Extract (BME) type 2 (Trevigen, 3533‐010‐02), and maintained in breast cancer organoid medium, which consisted of Advanced DMEM/F12 supplemented with R‐spondin‐1, noggin, neuregulin, estradiol, HEPES, GlutaMAX, nicotinamide, N‐acetylcysteine, B‐27, A83‐01, primocin, SB‐202190, Y27632, FGF10, FGF7, and EGF (all from specified suppliers). After 3‐5 passages, the organoids were plated in a 384‐well plate, and NK cells were added to each well at a 1:1 ratio for 4 h incubation. Cytotoxicity was assessed using a CellTiter‐Glo cell viability assay (Promega). For fluorescence imaging, organoids were stained with propidium iodide (PI) to visualize dead cells (Meilunbio, #MB2920‐1). All clinical samples were approved by the Ethics Committee of FUSCC and by each patient via signed informed consent.

### 4.17 Patient‐Derived Tumor Fragment (PDTF)

PDTF models were generated by culturing freshly resected breast tumor tissues as intact fragments, thereby preserving native immune and stromal components ex vivo. PDTFs were used to evaluate the direct effects of platinum treatment on intratumoral NK cell activation within a preserved tumor microenvironment. Following drug treatment, NK cell activation markers were analyzed by flow cytometry. Tissue preparation and drug treatment of PDTF were conducted as described previously [80]. In brief, fresh tumor tissues from patients were collected, preserved in a storage solution, and transported to the laboratory on ice at 4°C. The tumor specimens were cut into multiple 1 × 1 mm pieces, with 6–8 pieces placed in a cryovial, and cryopreservation solution (1 mL FBS supplemented with 10% dimethylsulfoxide) was added. Based on the pathological results of the tumor tissue, specimens from patients clinically typed as HR^+^/HER2^−^ were selected and resuscitated. Tumor culture medium was prepared by mixing DMEM with 1 mm sodium pyruvate, 1× MEM non‐essential amino acids, 2 mM L‐glutathione, 10% fetal bovine serum, and 1% penicillin–streptomycin. Collagen I and sodium bicarbonate were added to the medium on ice, followed by the formation of an artificial extracellular matrix. This matrix was added to the wells at 40 µL per well and allowed to solidify for 30 min. Revived tissue fragments were placed on the matrix, and an additional 40 µL of matrix was added, allowing it to solidify for another 30 min. Tumor culture medium supplemented with the control and drug treatments was added at 120 µL per well, with eight replicates. The plates were then incubated at 37°C with 5% CO2 for 48 h. For flow cytometry analysis, PDTFs were pooled (eight PDTFs were used per condition) and dissociated into single‐cell suspensions by enzymatic digestion in digestion mix (RPMI 1640 medium supplemented with 1% penicillin–streptomycin, 12.6 µg/mL Pulmozyme (Roche), and 1 mg/mL collagenase type IV (Sigma‐Aldrich)) for 1 h at 37°C and under slow rotation. Samples were then filtered over a 100 µm filter mesh and stained with activation markers. Flow cytometry acquisition typically yielded approximately 0.5 × 10^5^ to 0.5 × 10^6^ total events per pooled sample. Only samples with at least 1 × 10^4^ viable CD45^+^ events were included for analysis. All clinical samples were approved by the Ethics Committee of FUSCC and by each patient via signed informed consent.

### 4.18 In Vivo Models

Orthotopic syngeneic breast cancer models were established by implanting murine HR^+^ breast cancer cell lines (67NR or EMT6) into the mammary fat pads of immunocompetent BALB/c mice, allowing assessment of NK cell contributions to anti‐PD‐(L)1 therapy and its combination with platinum agents within an intact immune system. Briefly, 1 × 10^6^ 67NR cells or 0.5 × 10^6^ EMT6 cells were injected into the fourth mammary fat pad of female BALB/c mice. NK cells were selectively depleted by intraperitoneal administration of 10 µL anti–asialo‐GM1 per mouse on days −1, 6, and 13 relative to tumor implantation. Once tumors reached approximately 50–100 mm^3^, mice were treated with anti–PD‐1 monoclonal antibody (10 mg/kg) or the corresponding isotype control, administered twice weekly. Platinum‐based chemotherapy was delivered concomitantly, with carboplatin (25 mg/kg) or cisplatin (3 mg/kg) administered twice weekly. Mice were euthanized when the longest tumor diameter reached 20 mm or tumor volume exceeded 2000 mm^3^, after which peripheral blood, spleen, and tumors were surgically excised for downstream analyses. NK cell depletion efficiency was verified by flow cytometry of CD45^+^CD3^−^CD49b^+^ NK cells in spleen and peripheral blood samples. All animal experiments were performed in accordance with protocols approved by the Institutional Animal Care and Use Committee (IACUC) of Shanghai Chengxi Biotechnology Co., Ltd. (CX052307125). Body weight data for all in vivo experiments have been recorded to monitor systemic toxicity in Table S7.

### 4.19 Statistical Analysis

Statistical analysis was performed using R software (version 4.4.2) and GraphPad (version 8.0.1). Two‐way ANOVA was used to analyze the variance between the two growth curves. Wilcoxon test and Student's *t*‐test were used to compare the data between the two groups. A two‐sided *p* < 0.05 was considered statistically significant. *p < 0.05, **p < 0.01, ***p < 0.001; ns, not significant.

## Author Contributions

Conceptualization: Z.M.S., Y.Z.J.; Methodology: Z.M.S., Y.Z.J., M.H.S.; Experiments: Y.Y.C., Y.F.Z., Q.X., X.J., Y.X.Z., T.F.; Visualization: Y.Y.C., Y.F.Z., X.J., Q.X.; Writing – original draft: Y.Y.C., X.J.; Writing – review & editing: Z.M.S., Y.Z.J., M.H.S.

## Conflicts of Interest

The authors declare no conflicts of interest.

## Supporting information




**Supporting File**: advs74202‐sup‐0001‐SuppMat.docx

**Supporting Table**: advs74202‐sup‐0002‐TableS1‐S6.xlsx.

**Supporting Table**: advs74202‐sup‐0003‐TableS7.xlsx.

## Data Availability

The data generated in this study are available upon request from the corresponding author. The multi‐omics data from FUSCC re‐analyzed here are available in the Genome Sequence Archive (GSA) database under accession codes PRJCA017539 (https://ngdc.cncb.ac.cn/bioproject/browse/PRJCA017539). The processed scRNA‐Seq data of the FUSCC HR^+^/HER2^−^ immunotherapy cohort are available in the National Omics Data Encyclopedia under accession codes OEP00006564 (https://www.biosino.org/node/project/detail/OEP00006564). The bulk RNA‐seq data of platinum‐treated NK cells are available in the Sequence Read Archive (SRA) database under accession code PRJNA1272024. Bulk RNA‐seq data of the I‐SPY2 ICI therapy cohort were available from GSE173839 and GSE194040. TCGA data were downloaded from the cBioPortal website (www.cbioportal.org).
