# Supplementary material for: NK Cell Activation by Platinum Boosts Immunotherapy in HR+/HER2− Breast Cancer
Source: Adv Sci (Weinh). 2026 Feb 15;13(20):e18978. doi: 10.1002/advs.202518978 (PMC13067780; doi:10.1002/advs.202518978)
Supplement: Supplementary file 1 — Supporting File: advs74202‐sup‐0001‐SuppMat.docx [file ADVS-13-e18978-s001.docx]

Supporting Information

**NK Cell Activation by Platinum Boosts Immunotherapy in HR^+^/HER2^-^ Breast Cancer**

*Yi-Yu Chen^1†^, Yi-Fan Zhou^1†^, Xuan Qi^1†^, Ya-Xin Zhao^1^, Tong Fu^1^, Xi Jin^1*^, Minhong Shen^1*^, Yi-Zhou Jiang^1*^, Zhi-Ming Shao^1*^*

Includes: Figures S1 to S8 and Tables S1 to S7

S7 see Supplementary Table S7


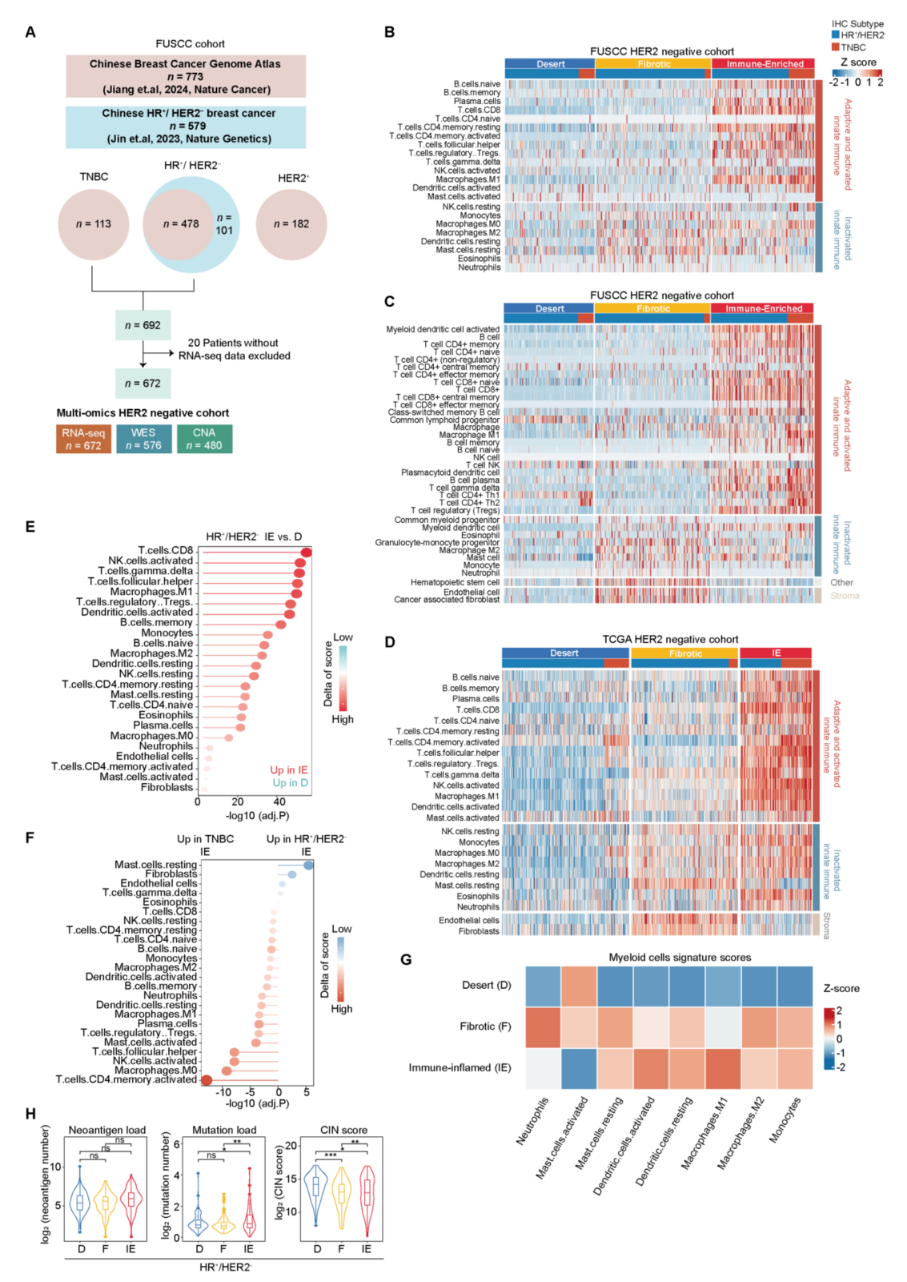


**Figure S1. Characterization of the tumor microenvironment in HR^+^/HER2^-^ breast cancer, related to Figure 1.** **(A)** Flowchart of the HER2 negative multi-omics cohort. **(B)** CIBERSORT-estimated cell proportion of 22 types of immune cells among the TME subtypes. Cell abundance was normalized across samples. **(C)** xCell-estimated cell proportion of 36 types of microenvironment cells among the TME subtypes. Cell abundance was normalized across samples. **(D)** K-means clustering of HER2 negative breast cancer from TCGA cohort based on the estimated abundance of 24 microenvironment cell subsets calculated by ssGSEA method. **(E)** Differential signatures between the Immune-Enriched (IE) and Desert (D) TME subtypes of HR^+^/HER2^-^ breast cancer. **(F)** Differential signatures between the Immune-Enriched (IE) subtypes of HR^+^/HER2^-^ breast cancer and TNBC. **(G)** Distribution of myeloid cell subpopulations across distinct TME subtypes. **(H)** Comparison of neoantigen load, mutation load, and chromosomal instability (CIN) score among the TME subtypes in HR^+^/HER2^-^ breast cancer. The central line of each boxplot within violin plots represents the median value, and the lower and upper hinges indicate the 25th and 75th percentiles, respectively. *P* values were from the two-sided unpaired Wilcoxon test.


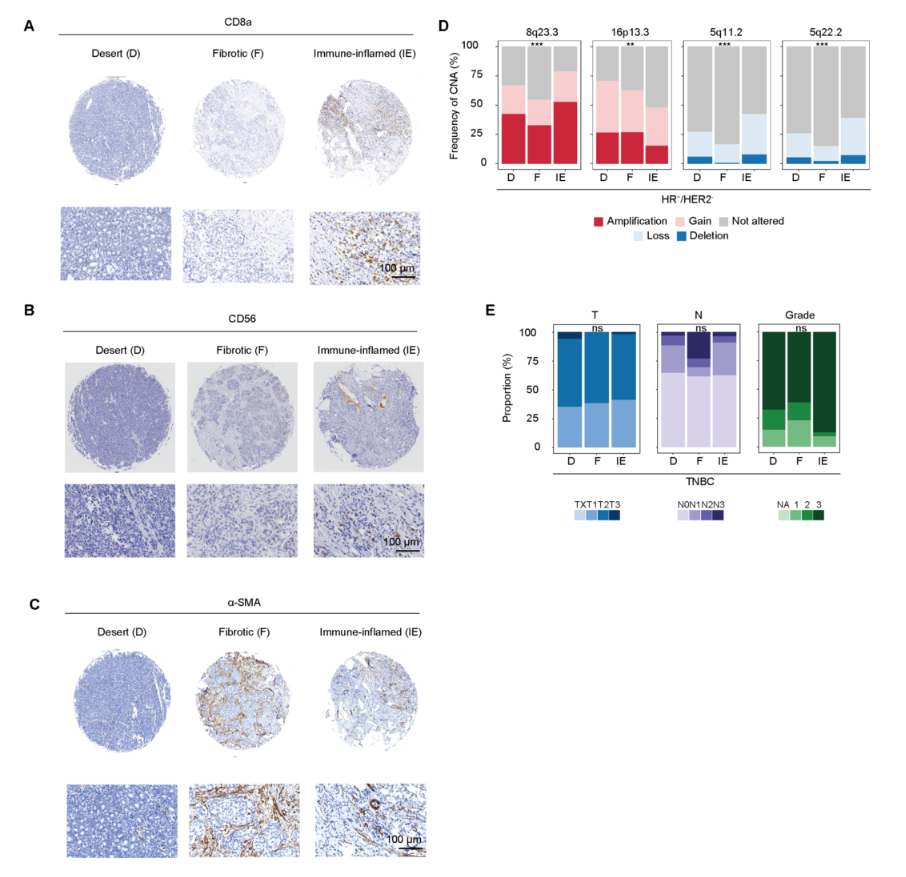


**Figure S2. Genomic and clinicopathological characteristics of breast cancer microenvironment subtypes, related to Figure 1. (A)** Representative immunohistochemistry staining of CD8a among the HR^+^/HER2^-^ TME subtypes. **(B)** Representative immunohistochemistry staining of CD56 among the HR^+^/HER2^-^ TME subtypes. **(C)** Representative immunohistochemistry staining of α-SMA among the HR^+^/HER2^-^ TME subtypes. **(D)** The association of copy number alterations (CNA) with HR^+^/HER2^-^ TME subtypes. **(E)** Distribution of tumor size staging, lymph node staging, and histological grading among the triple-negative breast cancer (TNBC) TME subtypes.


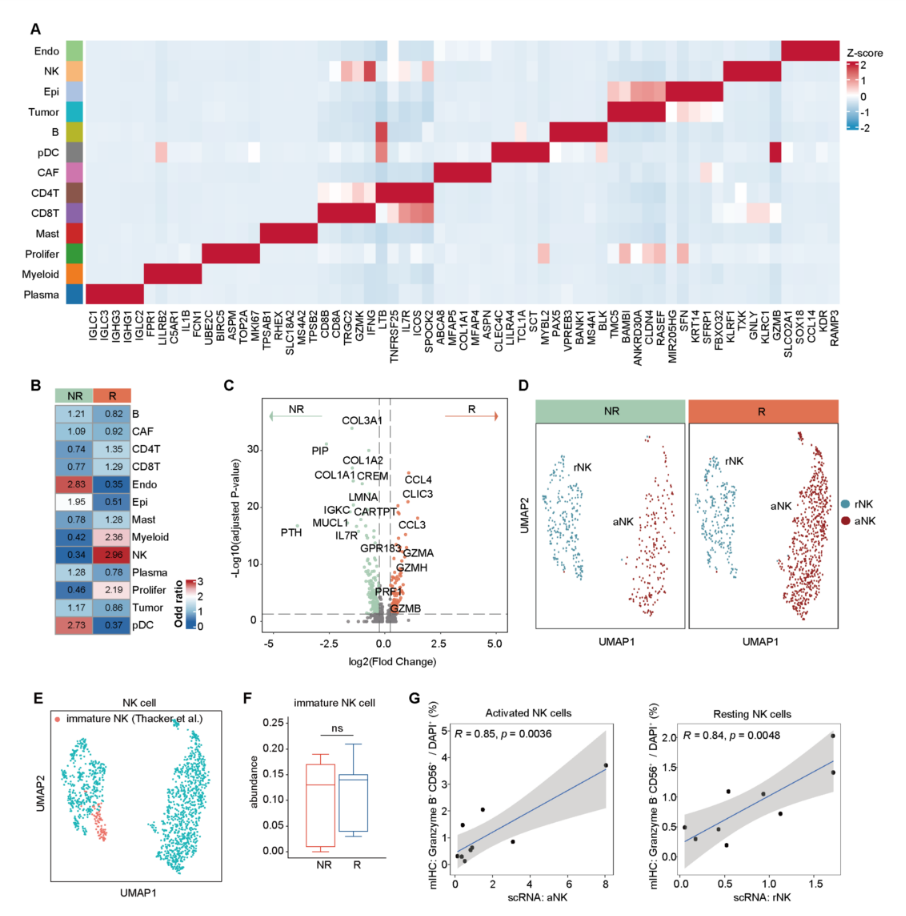


**Figure S3. Single-cell analysis reveals an association between activated NK cells and response to neoadjuvant immunotherapy, related to Figure 2. (A)** The expression patterns of signature genes in distinct cell clusters. **(B)** The distribution preferences of each cell subset between the Responder (R) and Non-Responder (NR) groups estimated by the STARTRAC method. **(C)** Volcano plots showing differentially expressed genes in NK cells from the different efficacy groups. **(D)** UMAP visualization of the resting NK cells (rNK) and activated NK cells (aNK) subsets in the different efficacy groups. **(E)** UMAP visualization identifying immature NK cells (red) in the HR^+^/HER2^-^ tumor microenvironment, based on established gene signatures by Thacker et al. **(F)** The abundance of infiltrating immature NK cells was compared between immunotherapy non-responders (NR) and responders (R) in the HR^+^/HER2^-^ cohort. **(G)** Correlation between the abundance of NK cell subsets quantified by single-cell RNA (scRNA) and their corresponding proportions measured by multiplex immunohistochemistry (mIHC).


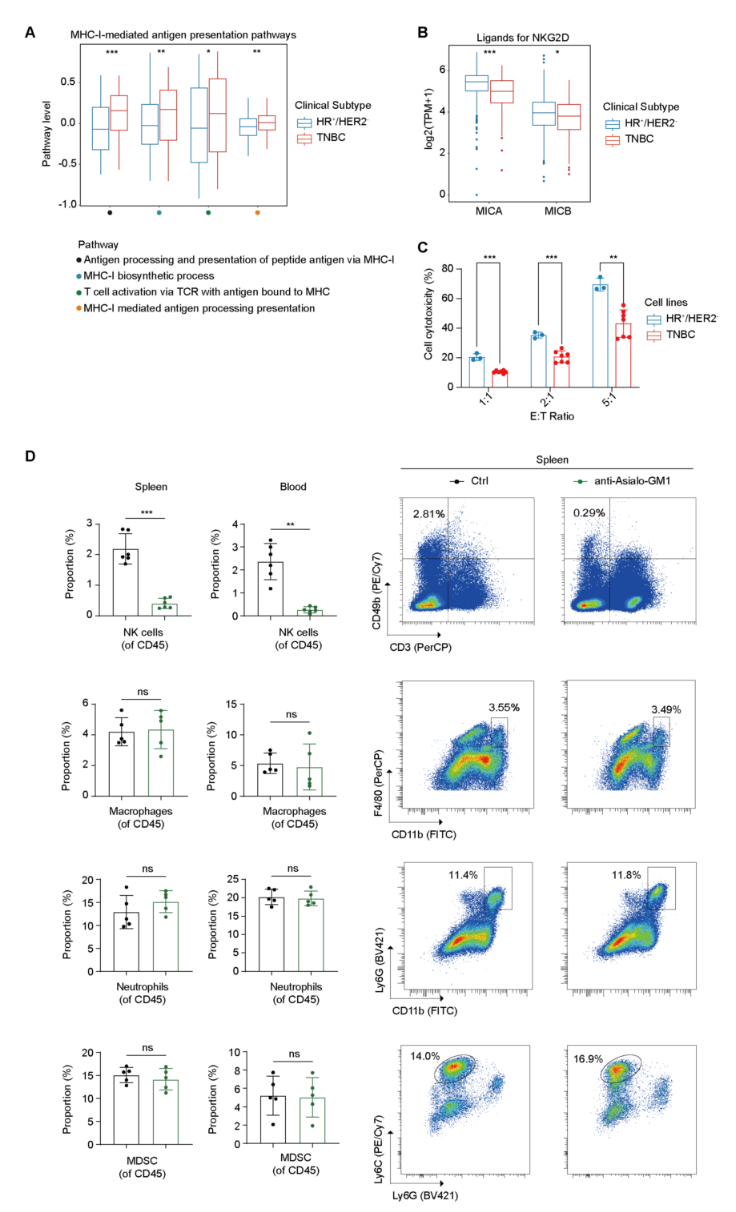


**Figure S4. HR^+^/HER2^-^ breast cancer shows enhanced susceptibility to NK cell-mediated cytotoxicity, related to Figure 3. (A)** Activation levels of MHC-I-mediated antigen presentation pathways in HR^+^/HER2^-^ and TNBC subtypes. The central line of each boxplot represents the median value, and the lower and upper hinges indicate the 25th and 75th percentiles, respectively. *P* values were from the two-sided unpaired Wilcoxon test. **(B)** Expression analysis of NK cell-related activating ligands in HR^+^/HER2^-^ and TNBC subtypes. MICA and MICB are the ligands for NKG2D. The central line of each boxplot represents the median value, and the lower and upper hinges indicate the 25th and 75th percentiles, respectively. *P* values were from the two-sided unpaired Wilcoxon test. **(C)** Differential susceptibility of HR^+^/HER2^-^ and TNBC cell lines to NK cell-mediated cytotoxicity. Two-tailed unpaired Student’s *t* test. **(D)** Flow cytometry representing the frequency of NK cells, macrophages, neutrophils, and myeloid-derived suppressor cells (MDSCs) in BALB/c mice treated with Control or anti-Asialo-GM1. Data are presented as mean ± SD. Two-tailed unpaired Student’s *t* test.


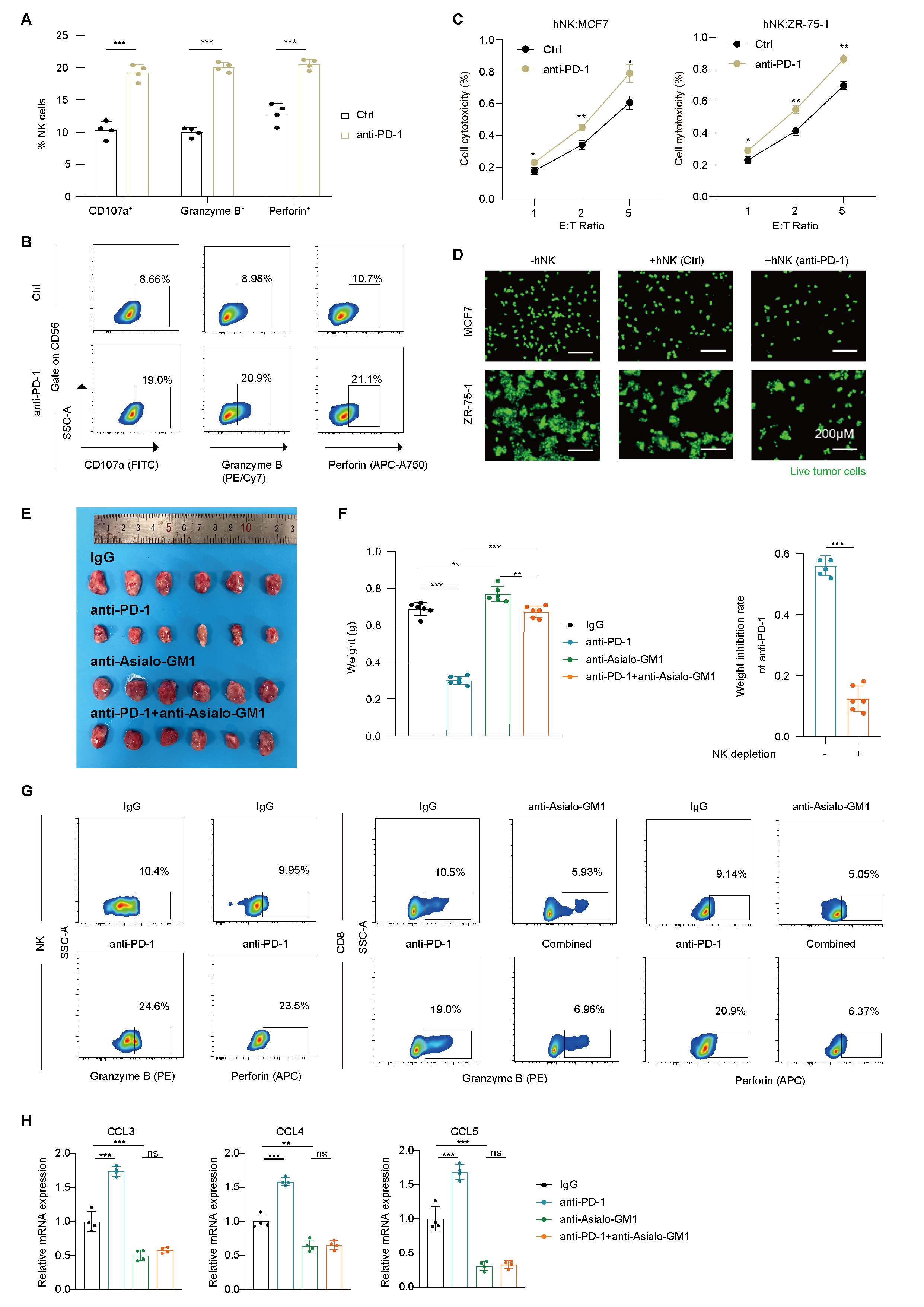


**Figure S5. Anti-PD-1 potentiates the cytotoxicity of NK cells, related to Figure 3. (A)** Statistical graph of flow cytometry representing the expression profile of functional markers in human peripheral blood-derived NK (hNK) cells treated with anti-PD-1. Data are presented as mean ± SD. Two-tailed unpaired Student’s *t* test. **(B)** Representative flow cytometry images representing the expression profile of functional markers in hNK cells treated with anti-PD-1. **(C)** Cytotoxicity of hNK cells treated with anti-PD-1 against MCF-7 and ZR-75-1 cells. Data are presented as mean ± SD. Two-tailed unpaired Student’s *t* test. **(D)** Representative images representing the cytotoxicity of hNK cells treated with anti-PD-1 against MCF-7 and ZR-75-1 cells. **(E)** Representative images of excised tumors from the indicated treatment groups in Figure 3H. **(F)** Tumor weight at the endpoint in mice treated with anti-PD-1 antibody and anti-Asialo-GM1 in the 67NR orthotopic tumor model (*n* = 6 per group). Data are presented as mean ± SD. Two-tailed unpaired Student’s *t* test. **(G)** Representative flow cytometry images representing the functional markers of CD8^+^ T cells and NK cells in EMT6 tumors. **(H)** qRT-PCR analysis of the relative transcript levels of CCL3, CCL4, and CCL5 in EMT6 tumors from mice treated with anti-PD-1 antibody and anti-Asialo-GM1. *n* = 4. Data are presented as mean ± SD. Two-tailed unpaired Student’s *t* test.

**
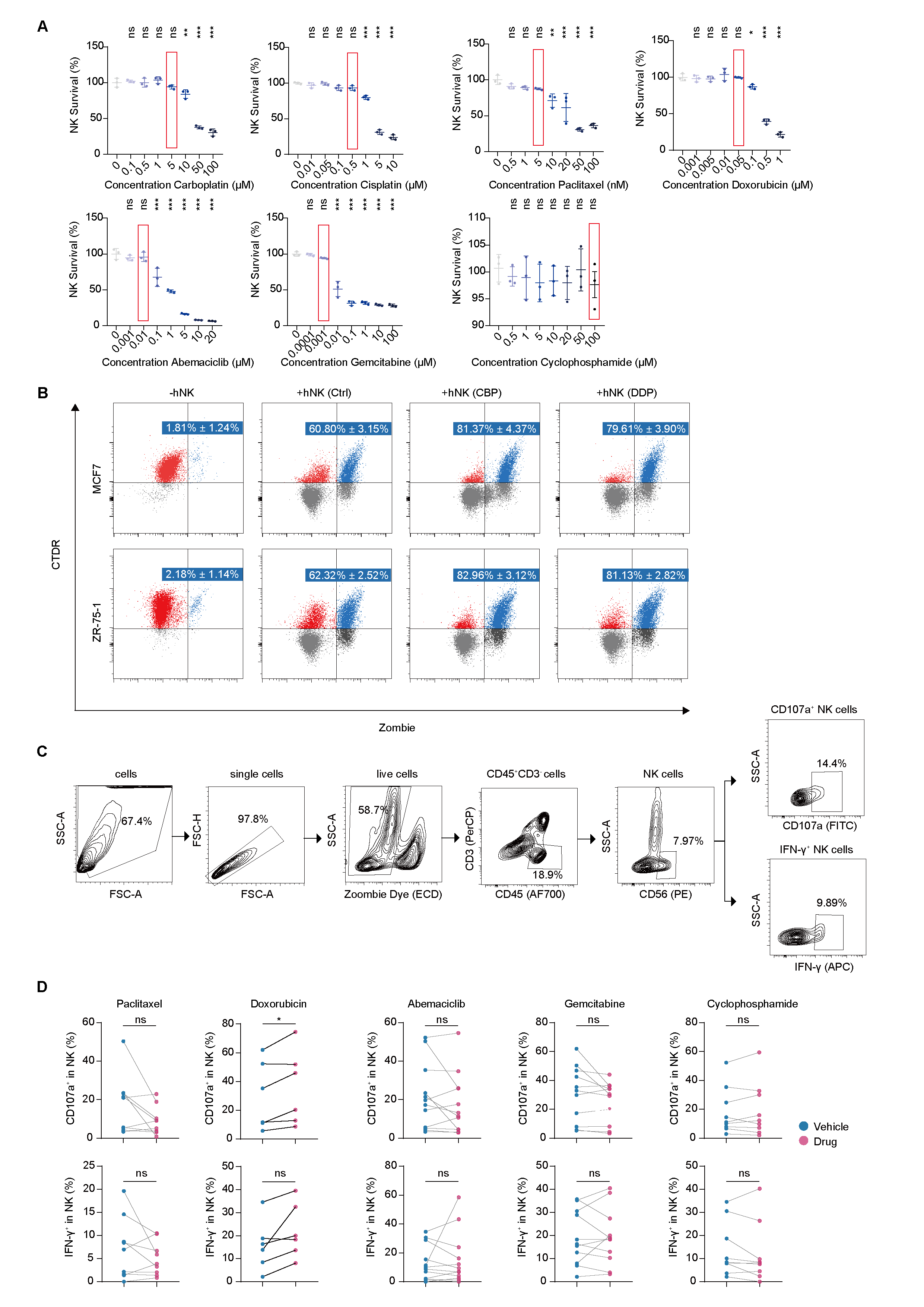
**

**Figure S6. Screening drugs for enhancing the function of NK cells, related to Figure 4. (A)** Survival rates of human peripheral blood-derived NK (hNK) cells treated with various agents. The red box represents the highest drug concentration that does not inhibit NK cell survival. *n* = 3. Data are presented as mean ± SD. Two-sided one-way ANOVA. **(B)** Flow cytometric quantification of NK cell-mediated MCF7 and ZR-75-1 tumor cell death following platinum pretreatment. Tumor cells were identified as CTDR^+^, and dead tumor cells were defined as CTDR^+^ Zombie^+^ after 4 h co-culture (E:T = 5:1). Representative plots are shown. Cytotoxicity was calculated as dead tumor cells / (live tumor cells + dead tumor cells) and annotated in the plots. *n* = 3. Data are presented as mean ± SD. **(C)** Gating strategies of Figure 4I. **(D)** Statistical graph of flow cytometry representing the expression profile of functional markers in NK cells from PDTF tissues treated with various drugs. Data are presented as mean ± SD. Two-tailed paired Student’s *t* test.


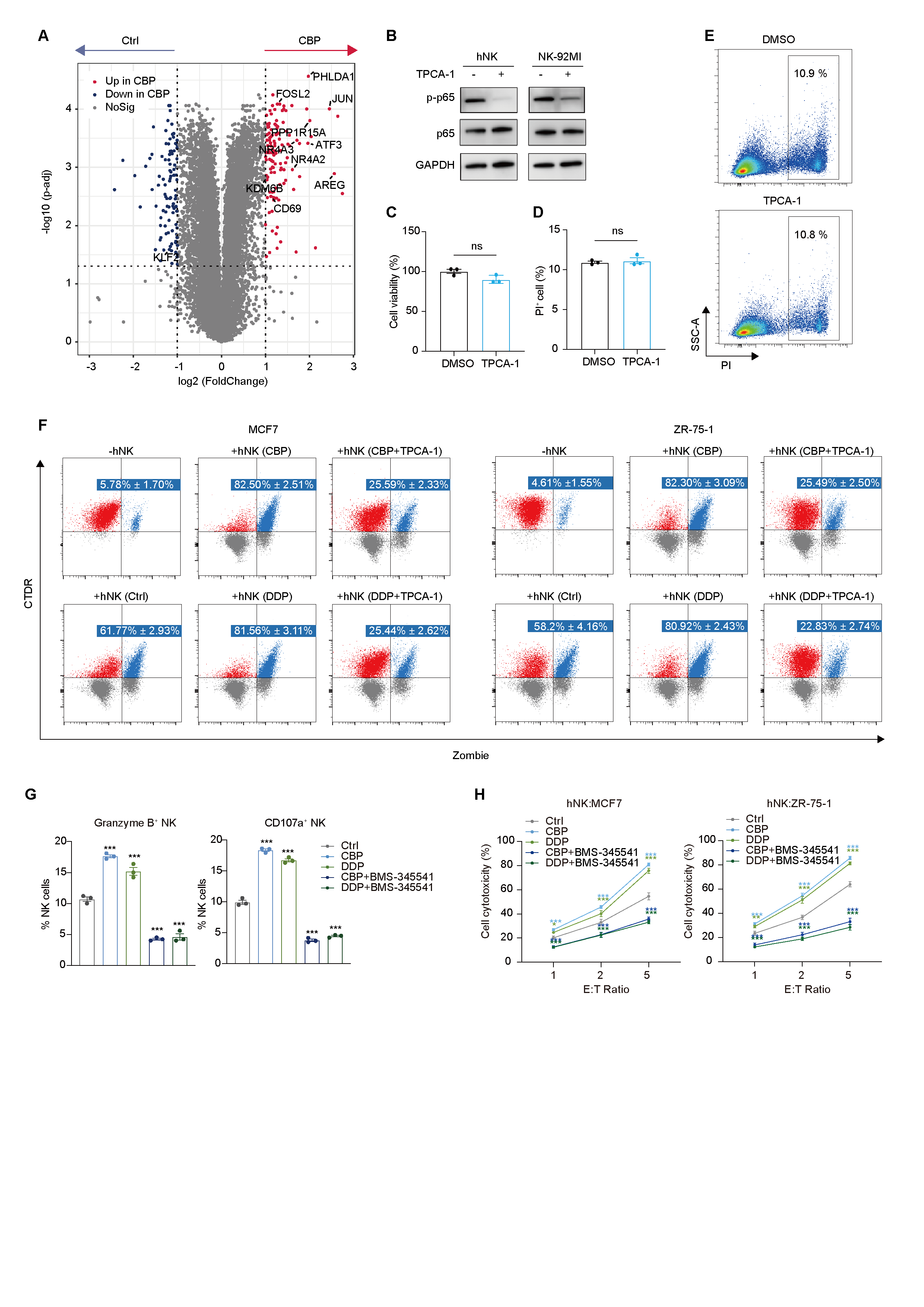


**Figure S7. Platinum induces NK cell activation via NF-κB pathway, related to Figure 5. (A)** Volcano plot of differentially expressed genes in NK-92MI cells treated with carboplatin (CBP). The leading-edge genes contributing to the NF-κB pathway enrichment are specifically highlighted. **(B)** Western blot analysis of the expression of p65 and p-p65 in control and TPCA-1-treated NK cells. **(C)** CCK-8 assay assessing the effect of TPCA-1 (2 μM) on hNK cell viability. *n* = 3. Data are presented as mean ± SD. Two-tailed unpaired Student’s *t* test. **(D)** Flow cytometric quantification of PI^+^ hNK cells following TPCA-1 treatment. *n* = 3. Data are presented as mean ± SD. Two-tailed unpaired Student’s *t* test. **(E)** Representative flow cytometry plots showing PI^+^ hNK cells after TPCA-1 treatment. **(F)** Flow cytometric quantification of NK cell-mediated MCF7 and ZR-75-1 tumor cell death following platinum and TPCA-1 pretreatment. Tumor cells were identified as CTDR^+^ and dead cells as CTDR^+^ Zombie^+^ after 4 h co-culture (E:T = 5:1). Representative plots are shown. Cytotoxicity was calculated as dead tumor cells / (live tumor cells + dead tumor cells) and annotated in the plots. *n* = 3. Data are presented as mean ± SD. **(G)** Flow cytometry analysis of the expression profile of functional markers in hNK cells co-treated with BMS-345541 and platinum drugs. Data are presented as mean ± SD. Two-tailed unpaired Student’s *t* test. *n* = 3. **(H)** Cytotoxicity of hNK cells treated with BMS-345541 and platinum drugs against MCF-7 and ZR-75-1 cells. *n* = 3. Data are presented as mean ± SD. Two-tailed unpaired Student’s *t* test.


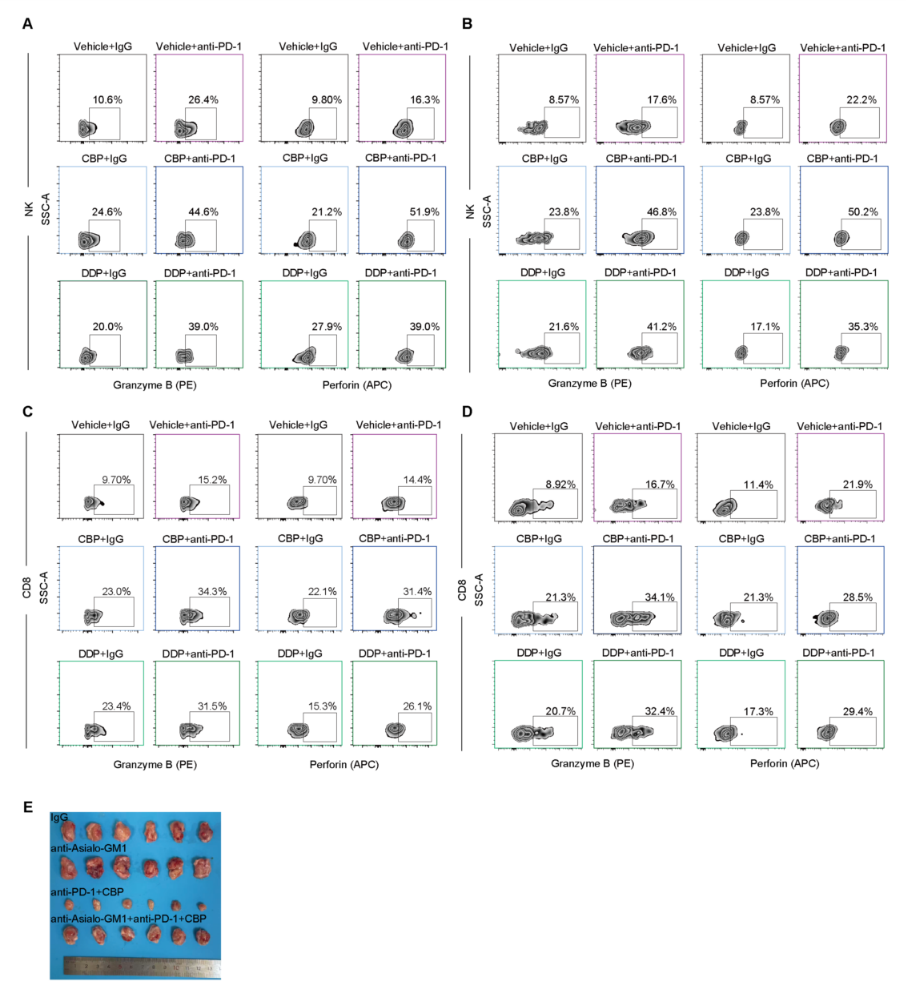


**Figure S8. Platinum enhanced the efficacy of immunotherapy in HR^+^/HER2^-^ breast cancer, related to Figure 6.**  **(A)** Representative flow cytometry images representing the functional markers of NK cells in 67NR tumors. **(B)** Representative flow cytometry images representing the functional markers of NK cells in EMT6 tumors. **(C)** Representative flow cytometry images representing the functional markers of CD8^+^ cells in 67NR tumors. **(D)** Representative flow cytometry images representing the functional markers of CD8^+^ cells in EMT6 tumors. **(E)** Representative images of excised tumors from the indicated treatment groups in Figure 6F.

**Table S1. Patient information of scRNA-seq cohort.**

| **Patient study ID** | **Age** | **Gender** | **Histology** | **cT** | **cN** | **M** | **RCB** | **Efficacy** |
| --- | --- | --- | --- | --- | --- | --- | --- | --- |
| FUSCC_01 | 45 | Female | Invasive Micropapillary Carcinoma | 2 | 2 | 0 | 2 | R |
| FUSCC_02 | 53 | Female | Invasive Ductal Carcinoma | 2 | 0 | 0 | 3 | NR |
| FUSCC_03 | 40 | Female | Invasive Ductal Carcinoma | 2 | 3 | 0 | 1 | R |
| FUSCC_04 | 63 | Female | Invasive Ductal Carcinoma | 4 | 2 | 0 | 2 | R |
| FUSCC_05 | 48 | Female | Invasive Ductal Carcinoma | 4 | 1 | 0 | 3 | NR |
| FUSCC_06 | 56 | Female | Invasive Ductal Carcinoma | 2 | 1 | 0 | 3 | NR |
| FUSCC_07 | 38 | Female | Invasive Ductal Carcinoma | 3 | 1 | 0 | 3 | NR |
| FUSCC_08 | 52 | Female | Invasive Ductal Carcinoma | 2 | 1 | 0 | 3 | NR |
| FUSCC_09 | 39 | Female | Invasive Ductal Carcinoma | 2 | 3 | 0 | 3 | NR |
| FUSCC_10 | 43 | Female | Invasive Micropapillary Carcinoma | 2 | 2 | 0 | 2 | R |
| FUSCC_11 | 58 | Female | Invasive Ductal Carcinoma | 2 | 2 | 0 | 2 | R |

scRNA, Single-cell RNA sequencing; T, tumor size; N, lymph node; M, Metastasis; RCB, residual tumor burden; R, responder; NR, non-responder

**Table S2. Patient information of FUSCC immunotherapy validation cohort.**

| **Patient study ID** | **Gender** | **Histology** | **cT** | **cN** | **M** | **Efficacy** |
| --- | --- | --- | --- | --- | --- | --- |
| FUSCC_V01 | Female | Invasive Ductal Carcinoma | 2 | 1 | 0 | NR |
| FUSCC_V02 | Female | Invasive Ductal Carcinoma | 3 | 3 | 0 | R |
| FUSCC_V03 | Female | Invasive Ductal Carcinoma | 3 | 1 | 0 | NR |
| FUSCC_V04 | Female | Invasive Ductal Carcinoma | 3 | 2 | 0 | NR |
| FUSCC_V05 | Female | Invasive Ductal Carcinoma | 2 | 2 | 0 | NR |
| FUSCC_V06 | Female | Invasive Lobular Carcinoma | 4 | 1 | 0 | NR |
| FUSCC_V07 | Female | Invasive Ductal Carcinoma | 2 | 2 | 0 | R |
| FUSCC_V08 | Female | Invasive Ductal Carcinoma | 1 | 1 | 0 | R |
| FUSCC_V09 | Female | Invasive Ductal Carcinoma | 2 | 1 | 0 | NR |
| FUSCC_V10 | Female | Invasive Ductal Carcinoma | 0 | 0 | 0 | NR |
| FUSCC_V11 | Female | Invasive Ductal Carcinoma | 2 | 3 | 0 | NR |
| FUSCC_V12 | Female | Invasive Ductal Carcinoma | 0 | 0 | 0 | R |
| FUSCC_V13 | Female | Invasive Ductal Carcinoma | 0 | 0 | 0 | R |
| FUSCC_V14 | Female | Invasive Ductal Carcinoma | 0 | 0 | 0 | R |
| FUSCC_V15 | Female | Invasive Ductal Carcinoma | 0 | 0 | 0 | NR |
| FUSCC_V16 | Female | Invasive Ductal Carcinoma | 2 | 1 | 0 | NR |
| FUSCC_V17 | Female | Invasive Micropapillary Carcinoma | 4 | 2 | 0 | R |
| FUSCC_V18 | Female | Invasive Ductal Carcinoma | 0 | 0 | 0 | NR |
| FUSCC_V19 | Female | Invasive Ductal Carcinoma | 0 | 0 | 0 | NR |
| FUSCC_V20 | Female | Invasive Ductal Carcinoma | 3 | 1 | 0 | R |
| FUSCC_V21 | Female | Invasive Ductal Carcinoma | 2 | 0 | 0 | NR |
| FUSCC_V22 | Female | Invasive Ductal Carcinoma | 4 | 1 | 0 | NR |
| FUSCC_V23 | Female | Invasive Ductal Carcinoma | 4 | 1 | 0 | NR |
| FUSCC_V24 | Female | Invasive Ductal Carcinoma | 2 | 1 | 0 | R |

T, tumor size; N, lymph node; M, Metastasis; R, responder; NR, non-responder

**Table S3. Signatures of steps in Cancer-Immunity Cycle**

| **Signature** | **Step** | **Genes** |
| --- | --- | --- |
| eat_me | Antigen presentation | SLAMF7,MAC1,CALR |
| dont_eat_me | Antigen presentation | CD47,PD-L1,LILRB2,SIRPA,PD-1 |
| APC_activating | Antigen presentation | FLT3LG,CSF2,CD40,CD40LG |
| cytokines_proinflame | Antigen presentation | TNF,IL1B,IL1A,IFNA2,IFNB1,IL2,IL12 |
| cytokines_inhib | Antigen presentation | IL10,IL4,IL13 |
| cGAS-STING | Antigen presentation | CGAS,TMEM173,IRF3 |
| Toll-like receptors | Antigen presentation | MYD88,TICAM1,TLR1,TLR10,TLR2,TLR3,TLR4,TLR5,TLR6,TLR7,TLR8,TLR9 |
| RIG-like receptors | Antigen presentation | DDX58,IFIH1,MAVS |
| C-type lectin receptors | Antigen presentation | CLEC7A,CLEC6A,CLEC4E,CD209,CLEC10A |
| Nod-like receptors | Antigen presentation | NLRP3,NLRP6,NLRP12,AIM2,PYCARD |
| Traffic_B cell | Trafficking | CXCR5,CXCL13 |
| Traffic_CD4 T cell | Trafficking | CCL19,CX3CL1,CXCL16 |
| Traffic_CD8 T cell | Trafficking | CCR5,CXCR3,CXCL10,CXCL9,CCL20,CXCL11,CX3CL1,CXCL16 |
| Traffic_Dendritic cell | Trafficking | CCR7,CCL3,CCL4,CCL5,CCL21 |
| Traffic_Macrophage | Trafficking | CSF1,CCL2,CCL3,CCL4,CCL5 |
| Traffic_NK cell | Trafficking | CXCR3,CXCL10,CXCL9,CCL3,CCL4,CCL5,CXCL11,CX3CL1 |
| Angiogenesis | Infiltration | ANGPT,ANGPT2,CDH5,CXCL5,CXCL8,CXCR2,FLT1,KDR,PDGFC,PGF,TEK,VEGFA,VEGFB,VEGFC,VWF |
| Matrix | Infiltration | COL11A1,COL1A1,COL1A2,COL3A1,COL4A1,COL5A1,ELN,FN1,LAMA3,LAMB3,LAMC2,LGALS7,LGALS9,TNC,VTN |
| CAF | Infiltration | ACTA2,CD248,COL11A1,COL1A1,COL1A2,COL5A1,COL6A1,COL6A2,COL6A3,CXCL12,FAP,FBLN1,FGF2,FN1,LRP1,LUM,MFAP5,MMP2,MMP3,PDGFRA,PDGFRB |
| mCAF | Infiltration | ACTA2,TAGLN,MMP11,MYL9,HOPX,POSTN,TPM1,TPM2 |
| TGF-β | Infiltration | TGFBR1,SMAD7,TGFB1,SMURF2,SMURF1,BMPR2,SKIL,SKI,ACVR1,PMEPA1,NCOR2,SERPINE1,JUNB,SMAD1,SMAD6,PPP1R15A,TGIF1,FURIN,SMAD3,FKBP1A,MAP3K7,BMPR1A,CTNNB1,HIPK2,KLF10,BMP2,ENG,APC,PPM1A,XIAP,CDH1,ID1,LEFTY2,CDKN1C,TRIM33,RAB31,TJP1,SLC20A1,CDK9,ID3,NOG,ARID4B,IFNGR2,ID2,PPP1CA,SPTBN1,WWTR1,BCAR3,THBS1,FNTA,HDAC1,UBE2D3,LTBP2,RHOA |
| HLA I | Recognition | HLA-A,HLA-B,HLA-C,TAP1,TAP2,B2M |
| HLA II | Recognition | HLA-DPA1,HLA-DPB1, HLA-DQA1,HLA-DQA2,HLA-DQB1,HLA-DQB2,HLA-DRB1,HLA-DRB5 |
| Inhibitory | Recognition | PDCD1,CTLA4,HAVCR2,BTLA,LAG3,TIGIT,ADORA2A,VSIR,CD276,CD274,PDCD1LG2,CD80,CD86,LGALS9,TNFRSF14,PVR |
| Stimulatory | Recognition | TNFRSF4,CD154,ICOS,CD27,TNFRSF18,TNFRSF9,CD226,TNFSF4,CD40,ICOSLG,CD70,TNFSF18,TNFSF9,NECTIN2 |
| CYT | Killing | GZMA,PRF1 |
| IFNg-6 | Killing | IDO1,CXCL10,CXCL9,HLA-DRA,IFNG,STAT1 |
| IFNg expanded immune 18 | Killing | CD3D,IL2RG,NKG7,CIITA,HLA-E,CD3E,CXCR6,CCL5,LAG3,TAGAP,GZMK,CD2,IDO1,CXCL10,HLA-DRA,STAT1,CXCL13,GZMB |
| Effector T-cell | Killing | GZMA,GZMB,PRF1,IFNG,EOMES,CD8A |
| IFNg/Effector T-cell | Killing | CD8A,GZMA,GZMB,IFNG,EOMES,CXCL9,CXCL10,TBX21 |

**Table S4. Antibodies for western blot, immunohistochemistry, flow cytometry, and in-vivo experiments.**

| **Name** | **Company** | **Use** | **Dilution** | **Cat#** | **clone** |
| --- | --- | --- | --- | --- | --- |
| CD8a | Servicebio | IHC | 1:1500 | GB12068 | / |
| αSMA | Servicebio | IHC | 1:200 | GB13044 | / |
| Mo&Rb | GeneTech | IHC | / | GK6007 | / |
| Granzyme B | Abcam | mIHC | 1:3000 | ab255598 | / |
| CD56 | Abcam | mIHC | 1:2000 | ab75813 | / |
| p65 | Cell Signaling | WB | 1:1000 | 8242 | / |
| p-p65 | Cell Signaling | WB | 1:1000 | 3033 | / |
| GAPDH | Proteintech | WB | 1:10000 | 10494-1-AP | / |
| HRP-linked anti-rabbit antibodies | Proteintech | WB | 1:5000 | SA00001-2 | / |
| TruStain FcX™ (anti-mouse CD16/32) Antibody | BioLegend | FC | 1.0 µg per million cells in 100 µl | 101320 | 93 |
| APC/Fire™ 750 anti-mouse CD45 Antibody | BioLegend | FC | 0.25 µg per million cells in 100 µl | 103154 | 30-F11 |
| FITC anti-mouse CD8a | BioLegend | FC | 1.0 µg per million cells in 100 µl | 100706 | 53-6.7 |
| PerCP/Cyanine5.5 anti-mouse CD3 Antibody | BioLegend | FC | 1.0 µg per million cells in 100 µl | 100218 | 17A2 |
| PE/Cyanine7 anti-mouse CD49b | BioLegend | FC | 0.5 µg per million cells in 100 µl | 108922 | DX5 |
| PE anti-human/mouse Granzyme B | BioLegend | FC | 5 µl per million cells in 100 µl | 372208 | QA16A02 |
| APC anti-mouse Perforin Antibody | BioLegend | FC | 0.5 µg per million cells | 154404 | S16009B |
| PE/Cyanine7 anti-human/mouse Granzyme B Recombinant Antibody | BioLegend | FC | 5 µl per million cells in 100 µl | 372213 | QA16A02 |
| APC/Fire™ 750 anti-human Perforin Antibody | BioLegend | FC | 5 µl per million cells in 100 µl | 353317 | [B-D48](https://www.biolegend.com/en-gb/search-results?Clone=B-D48) |
| FITC anti-human CD107a (LAMP-1) Antibody | BioLegend | FC | 5 µl per million cells in 100 µl | 328606 | H4A3 |
| APC anti-human IFN-γ Antibody | BioLegend | FC | 5 µl per million cells in 100 µl | 502511 | [4S.B3](https://www.biolegend.com/en-gb/search-results?Clone=4S.B3) |
| Alexa Fluor® 700 anti-human CD45 Antibody | BioLegend | FC | 1.0 µg per million cells in 100 µl | 304024 | [HI30](https://www.biolegend.com/en-gb/search-results?Clone=HI30) |
| PE anti-human CD56 (NCAM) Antibody | BioLegend | FC | 5 µl per million cells in 100 µl | 318306 | HCD56 |
| Zombie Aqua™ Fixable Viability Kit | BioLegend | FC | 1 µl per million cells in 100 µl | 423101 | / |
| Zombie Red™ Fixable Viability Kit | BioLegend | FC | 1 µl per million cells in 100 µl | 423110 | / |
| PerCP/Cyanine5.5 anti-mouse F4/80 | BioLegend | FC | 1.0 µg per 106 cells in 100 µl | 123128 | BM8 |
| FITC anti-mouse/human CD11b | BioLegend | FC | 0.25 µg per 106 cells in 100 µl | 101206 | M1/70 |
| Brilliant Violet 421™ anti-mouse Ly-6G | BioLegend | FC | 0.5 µg per million cells in 100 µl | 127627 | 1A8 |
| PE/Cyanine7 anti-mouse Ly-6C | BioLegend | FC | 0.06 µg per million cells in 100 µl | 128017 | HK1.4 |
| InVivoMab anti-mouse PD-1 | BioXCell | In vivo | / | BE0146 | / |
| InVivoMab rat IgG2a isotype control | BioXCell | In vivo | / | BE0089 | / |
| Ultra-LEAF™ Purified anti-Asialo-GM1 Antibody | BioLegend | In vivo | / | 146002 | / |

IHC, immunohistochemistry; mIHC, multiplex immunohistochemistry; WB, western blot; FC, flow cytometry.

**Table S5. Primers for RT-qPCR**

| **Primer** | **Species** | **Sequence** |
| --- | --- | --- |
| CCL3-Forward | Human | ACTTTGAGACGAGCAGCCAGTG |
| CCL3-Reverse | Human | TTTCTGGACCCACTCCTCACTG |
| CCL4-Forward | Human | GCTTCCTCGCAACTTTGTGGTAG |
| CCL4-Reverse | Human | GGTCATACACGTACTCCTGGAC |
| CCL5-Forward | Human | CCTGCTGCTTTGCCTACATTGC |
| CCL5-Reverse | Human | ACACACTTGGCGGTTCTTTCGG |
| GZMB-Forward | Human | CGACAGTACCATTGAGTTGTGCG |
| GZMB-Reverse | Human | TTCGTCCATAGGAGACAATGCCC |
| PRF1-Forward | Human | ACTCACAGGCAGCCAACTTTGC |
| PRF1-Reverse | Human | CTCTTGAAGTCAGGGTGCAGCG |
| IFNG-Forward | Human | GAGTGTGGAGACCATCAAGGAAG |
| IFNG-Reverse | Human | TGCTTTGCGTTGGACATTCAAGTC |
| ACTB-Forward | Human | CACCATTGGCAATGAGCGGTTC |
| ACTB-Reverse | Human | AGGTCTTTGCGGATGTCCACGT |
| CCL3-Forward | Mouse | ACTGCCTGCTGCTTCTCCTACA |
| CCL3-Reverse | Mouse | ATGACACCTGGCTGGGAGCAAA |
| CCL4-Forward | Mouse | ACCCTCCCACTTCCTGCTGTTT |
| CCL4-Reverse | Mouse | CTGTCTGCCTCTTTTGGTCAGG |
| CCL5-Forward | Mouse | CCTGCTGCTTTGCCTACCTCTC |
| CCL5-Reverse | Mouse | ACACACTTGGCGGTTCCTTCGA |
| ACTB-Forward | Mouse | CATTGCTGACAGGATGCAGAAGG |
| ACTB-Reverse | Mouse | TGCTGGAAGGTGGACAGTGAGG |

RT-qPCR, Quantitative Real-Time Polymerase Chain Reaction

**Table S6. Information of chemotherapeutics agents.**

| **Chemicals** | **Source** | **Identifiers** | **Solvent in vitro** | **Stock concentration** | **Working concentration** | **Final solvent** |
| --- | --- | --- | --- | --- | --- | --- |
| Cisplatin | MedChem | HY-17394 | DMF | 10 mM | 0.5 μM | 0.005% DMF |
| Carboplatin | Sigma-Aldrich | C2538 | water | 10 mM | 5 μM | - |
| Paclitaxel | Selleck Chemicals | S1150 | DMSO | 50 mM | 5 nM | 0.00001% DMSO |
| Doxorubicin | Selleck Chemicals | E2516 | water | 100 mM | 0.05 μM | - |
| Abemaciclib | Selleck Chemicals | S5716 | DMSO | 10 mM | 0.01 μM | 0.0001% DMSO |
| Gemcitabine | Selleck Chemicals | S1714 | DMSO | 100 mM | 0.001 μM | 0.00001% DMSO |
| Cyclophosphamide | MedChem | HY-17420 | DMSO | 300 mM | 100 μM | 0.033% DMSO |

DMF, N,N-Dimethylformamide; DMSO, Dimethyl sulfoxide
